# Targeting FROUNT with disulfiram suppresses macrophage accumulation and its tumor-promoting properties

**DOI:** 10.1038/s41467-020-14338-5

**Published:** 2020-01-30

**Authors:** Yuya Terashima, Etsuko Toda, Meiji Itakura, Mikiya Otsuji, Sosuke Yoshinaga, Kazuhiro Okumura, Francis H. W. Shand, Yoshihiro Komohara, Mitsuhiro Takeda, Kana Kokubo, Ming-Chen Chen, Sana Yokoi, Hirofumi Rokutan, Yutaka Kofuku, Koji Ohnishi, Miki Ohira, Toshihiko Iizasa, Hirofumi Nakano, Takayoshi Okabe, Hirotatsu Kojima, Akira Shimizu, Shiro Kanegasaki, Ming-Rong Zhang, Ichio Shimada, Hiroki Nagase, Hiroaki Terasawa, Kouji Matsushima

**Affiliations:** 1 Division of Molecular Regulation of Inflammatory and Immune Diseases, Research Institute for Biomedical Sciences (RIBS), Tokyo University of Science, Chiba, 278-0022 Japan; 2 Department of Molecular Preventive Medicine, Graduate School of Medicine, The University of Tokyo, Tokyo, 113-0033 Japan; 3 Department of Analytic Human Pathology, Nippon Medical School, Tokyo, 113-8602 Japan; 4 Department of Thoracic Disease, Chiba Cancer Center, Chiba, 260-8717 Japan; 5 Chiba Cancer Center Research Institute, Chiba, 260-8717 Japan; 6 Department of Anesthesiology, Tokyo Teishin Hospital, Tokyo, 102-8798 Japan; 7 Department of Structural BioImaging, Faculty of Life Sciences, Kumamoto University, Kumamoto, 862-0973 Japan; 8 Department of Cell Pathology, Graduate School of Medical Sciences, Kumamoto University, Kumamoto, 860-8556 Japan; 9 Graduate School of Pharmaceutical Sciences, The University of Tokyo, Tokyo, 113-0033 Japan; 10 Drug Discovery Initiative, The University of Tokyo, Tokyo, 113-0033 Japan; 11 Research Institute, National Center for Global Health and Medicine, Tokyo, 162-8655 Japan; 12 Department of Radiopharmaceutics Development, National Institutes for Quantum and Radiological Science and Technology, Chiba, 263-8555 Japan; 13 Present Address: Division of Molecular Regulation of Inflammatory and Immune Diseases, Research Institute for Biomedical Sciences (RIBS), Tokyo University of Science, Chiba, 278-0022 Japan; 14 Present Address: Department of Analytic Human Pathology, Nippon Medical School, Tokyo, 113-8602 Japan; 15 Present Address: Chiba Cancer Center Research Institute, Chiba, 260-8717 Japan; 16 Present Address: Department of Anesthesiology, Tokyo Teishin Hospital, Tokyo, 102-8798 Japan

**Keywords:** Cancer microenvironment, Cancer immunotherapy, Chemotaxis, High-throughput screening, Tumour immunology

## Abstract

Tumor-associated macrophages affect tumor progression and resistance to immune checkpoint therapy. Here, we identify the chemokine signal regulator FROUNT as a target to control tumor-associated macrophages. The low level *FROUNT* expression in patients with cancer correlates with better clinical outcomes. *Frount*-deficiency markedly reduces tumor progression and decreases macrophage tumor-promoting activity. FROUNT is highly expressed in macrophages, and its myeloid-specific deletion impairs tumor growth. Further, the anti-alcoholism drug disulfiram (DSF) acts as a potent inhibitor of FROUNT. DSF interferes with FROUNT-chemokine receptor interactions via direct binding to a specific site of the chemokine receptor-binding domain of FROUNT, leading to inhibition of macrophage responses. DSF monotherapy reduces tumor progression and decreases macrophage tumor-promoting activity, as seen in the case of *Frount*-deficiency. Moreover, co-treatment with DSF and an immune checkpoint antibody synergistically inhibits tumor growth. Thus, inhibition of FROUNT by DSF represents a promising strategy for macrophage-targeted cancer therapy.

## Abstract (web summary)

The cytoplasmic protein FROUNT can bind to chemokine receptors and enhance chemokine signalling. Here, the authors show that inhibiting FROUNT in macrophages either by knockdown of the gene or using the anti-alcoholism drug disulfiram, results in a reduction in tumour growth.

## Introduction

The tumor microenvironment consists of various immune and other cell types that contribute to tumor growth and malignancy, making it a promising target for cancer therapy^1^. Macrophages are one of the most abundant cell types in the tumor microenvironment^2^. Once recruited to the tumor site, macrophages are educated to display immunosuppressive or tumor-promoting phenotypes^3^. Accordingly, both the macrophage density and phenotype are associated with tumor malignancy and poor prognosis in patients with cancer^4^. Macrophages are recruited to and accumulate at the tumor site in response to chemoattractants^5^. The chemokine CCL2 was first identified in 1989 as a major chemoattractant for monocytes (macrophage progenitors) and macrophages^6,7^, and elevated expression of CCL2 and its receptor CCR2 correlates with a poor prognosis in patients with cancer^8–11^. In animal experiments, CCL2 was shown to be responsible for increased tumor growth and metastasis due to the accumulation of CCR2-expressing macrophages^11–13^. FROUNT (also known as NUP85) is a cytoplasmic protein that interacts with CCR2 and modulates the magnitude of chemotactic signals via activation of the PI3K-Rac-lamellipodium cascade^14^. FROUNT also binds to and promotes chemotactic signaling via CCR5^15^, another major chemokine receptor expressed on macrophages. Blockade of CCR2 or CCR5 has been shown to inhibit tumor progression in some animal models^11–13,16,17^. Although distinct in their chemokine usage, these receptors share a common binding region for FROUNT in the intracellular membrane-proximal C-terminal domain. The amino acid sequence of this region, as well as that of FROUNT, is highly conserved between mice and humans^14^, making it an attractive therapeutic target. Although several studies have demonstrated a role of FROUNT in inflammation^18,19^, its role in tumor progression remains unclear.

In this study, we show that FROUNT modulates tumor-associated macrophage responses and regulates tumor progression. Moreover, a retrospective analysis of patients with lung carcinoma indicated that a lower level of FROUNT expression is associated with a better prognosis. Studies using clinical specimens and *Frount-gfp* reporter mice showed that FROUNT is highly expressed in macrophages, and *Frount* deficiency in mice decreased macrophage accumulation at the tumor site and impaired the tumor-promoting activity of macrophages. Multi-step screening of a library of 131,200 compounds for inhibitors that block the interaction of FROUNT and CCR2/CCR5 by binding to FROUNT revealed disulfiram (DSF), a clinically approved drug for alcoholism, as a candidate potent inhibitor of FROUNT. Indeed, DSF reduced macrophage accumulation in the tumor, suppressed macrophage activity, increased the numbers of cytotoxic CD8^+^ T cells in the tumor when combined with the immune-checkpoint inhibitor anti-PD-1 antibody, and inhibited tumor growth and metastasis. Overall, our findings suggest that regulating tumor-promoting macrophages by targeting FROUNT may be a safe and effective approach in cancer therapy.

## Results

### FROUNT expression is associated with poor cancer prognosis

**Fig. 1:**
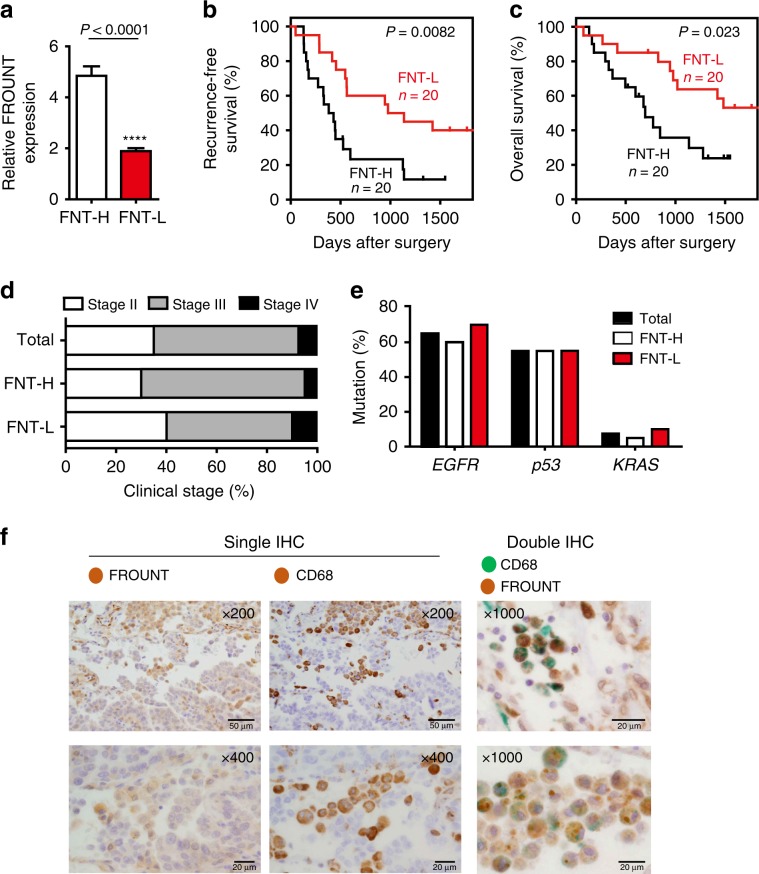
FROUNT expression is a risk factor for poor prognosis in patients with lung cancer. **a** FROUNT expression was measured in total RNA isolated from tumor specimens and patients were divided into *FROUNT*-high (*n* = 20) and *FROUNT*-low groups (*n* = 20). **b**, **c** Survival analysis of *FROUNT*-high (FNT-H) and -low (FNT-L) patients with lung cancer. Recurrence-free (**b**) and overall survival curves (**c**). **d**–**e** Clinical stages (**d**) and the prevalence of *EGFR*, *p53*, and *KRAS* mutations (**e**) were comparable between FNT-H and FNT-L groups. **f** Immunohistochemistry (IHC) of FROUNT and CD68 (brown), and double IHC of FROUNT (brown) and CD68 (green) in human lung adenocarcinoma. Representative images of tumors of a patient among 30 patient samples tested. Scale bars, 20 μm. ****P < 0.0001 by two-tailed, unpaired Student’s *t*-test. Error bars indicate s.e.m.

Considering the role of FROUNT as a common regulator of chemokine receptors CCR2 and CCR5, which have been implicated in tumor progression, we hypothesized that FROUNT expression levels affect clinical outcomes. Cox proportional-hazards regression analysis of the association between *FROUNT* mRNA levels and clinical outcomes in 40 patients with lung carcinoma revealed that *FROUNT* expression was a significant risk factor for recurrence (*P* = 0.0037) and survival (*P* = 0.015). When patients were divided into *FROUNT*-high (*n* = 20) and *FROUNT*-low groups (*n* = 20) (Fig. 1a), recurrence-free and total survival rates were significantly higher in the *FROUNT*-low group than the *FROUNT*-high group (Fig. 1b, c). *FROUNT* expression was independent of clinical stage (Fig. 1d) and other major prognostic factors such as the presence of *EGFR*, *p53*, or *KRAS* mutations (Fig. 1e). Even when compared separately between stage II and stages III + IV, the *FROUNT*-high group exhibited lower survival than the *FROUNT*-low group (Supplementary Fig. 1a). Similar results were obtained for another independent group of 31 patients with lung cancer (Supplementary Fig. 1b−e).

To confirm the prognostic significance of FROUNT in other human cancers, we examined data from the PRECOG (166 studies), PrognoScan (86 studies) and HumanProteinAtlas (17 studies) human transcriptome databases, and studies selected based on the criteria of FDR *q*-values (the *q*-value is a measure of the strength of an observed statistic with respect to FDR^20^) < 0.05 revealed that the patients with higher expression of FROUNT exhibit a poorer prognosis (Supplementary Fig. 2a-c). These analyses reinforce the correlation between high FROUNT expression and negative prognosis, suggesting that FROUNT is a prognostic marker for a range of human cancers.

### FROUNT deficiency impairs tumor growth and metastasis

Immunohistochemical staining of lung cancer specimens with an anti-FROUNT antibody showed that FROUNT is expressed in stromal cells, and highly expressed by CD68^+^ tumor-associated macrophages in particular (Fig. 1f). In human peripheral monocyte-derived macrophages, almost all CD68^+^ macrophages were found to be FROUNT-positive (Supplementary Fig. 3a). FROUNT expression as quantified by anti-FROUNT antibody staining correlated positively with *FROUNT*-mRNA expression (Supplementary Fig. 3b). Furthermore, there was a correlation between *FROUNT* mRNA expression and expression of the myeloid cell-related immunosuppressive markers CD204, PD-L1, and PD-L2 (Supplementary Fig. 3c). These findings suggest that *FROUNT* expression is mainly derived from CD68^+^ tumor-associated macrophages.

**Fig. 2:**
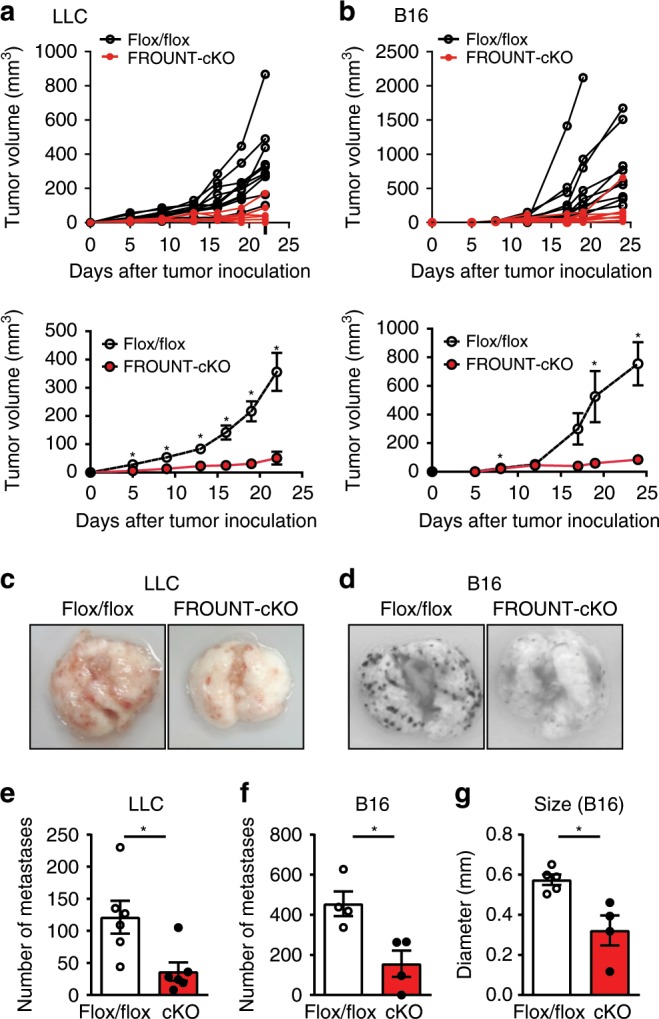
FROUNT deficiency in the host impaired tumor progression and metastasis. **a**, **b** Individual (upper graph) and combined (lower graph) tumor-growth kinetics in *Frount*-floxed (Flox/flox) or conditional knockout (FROUNT-cKO) mice inoculated subcutaneously with Lewis lung carcinoma (LLC) (Flox/flox: *n* = 12, cKO: *n* = 11) (**a**) or B16 melanoma (B16) cells (Flox/flox: *n* = 10, cKO: *n* = 10) (**b**). **c**–**g** Lung metastasis was examined by injecting LLC (**c** and **e**) or B16 (**d**, **f**, and **g**) tumor cells intravenously into Flox/flox or FROUNT-cKO mice. Representative images of tumor-bearing lungs (**c**, **d**); number (*n* = 6 for LLC and = 4 for B16) (**e, f**) and size (Flox/flox: *n* = 5, cKO: *n* = 4) (**g**) of visible metastatic nodules. *P < 0.05 by two-tailed, unpaired Student’s *t*-test. Error bars indicate s.e.m.

To determine the role of host FROUNT expression in tumor progression, we generated *Frount*-conditional knockout (FROUNT-cKO) mice (Supplementary Fig. 4a–e). In these mice, transplanted Lewis lung carcinoma (LLC) and B16 melanoma (B16) tumor growth was reduced compared to that in control mice (Fig. 2a, b), and metastatic nodule formation in the lungs after intravenous injection of tumor cells was reduced in both number and size (Fig. 2c–g). CAG^−^Cre expression had no effect on tumor metastasis in this model (Supplementary Fig. 4f, g). Together, these results demonstrate that FROUNT expression in the host is a key determinant of tumor progression.

### FROUNT deficiency reduces macrophage accumulation in tumor

**Fig. 3:**
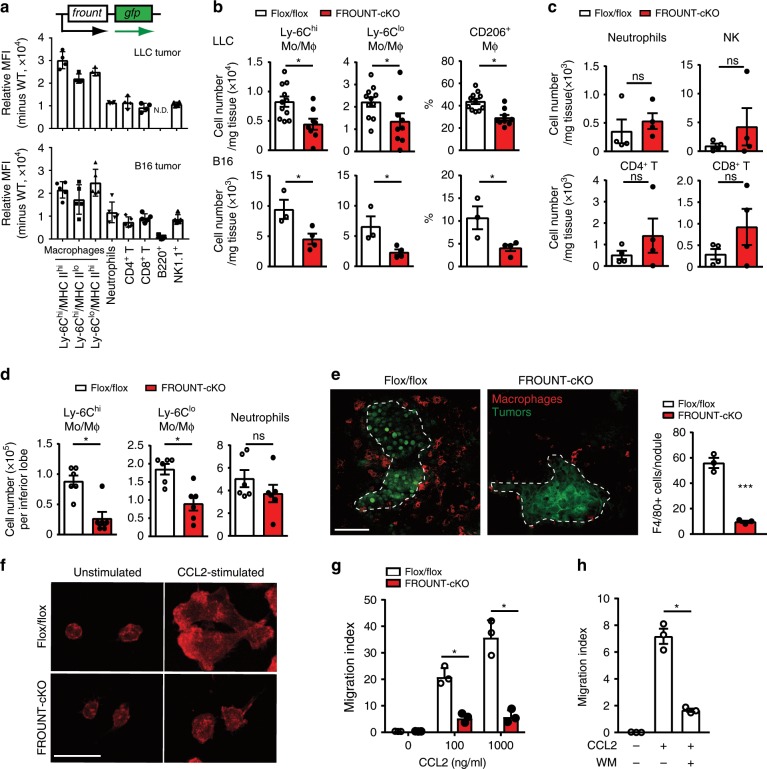
FROUNT deficiency impaired monocyte/macrophage accumulation at tumor sites. **a** FROUNT protein expression in LLC- or B16-tumor-infiltrating leukocyte subsets (monocytes/macrophages subsets; Ly-6C^hi^/MHC class II^hi^, Ly-6C^hi^/MHC class II^lo^ and Ly-6C^lo^/MHC class II^hi^, Neutrophils, CD4/8 T cells, B cells and NK cells) in FROUNT-GFP reporter mice as measured by flow cytometry (*n* = 4 for LLC and *n* = 5 for B16). **b** Numbers of tumor-associated Ly-6C^hi^ or Ly-6C^lo^ monocytes/macrophages (Mo/Mϕ) and proportion of CD206^+^ M2-type macrophages among total macrophages (CD45^+^CD11b^+^CD24^−^Ly-6G^−^ cells in leukocyte gate) in subcutaneous LLC (Flox/flox: *n* = 11, cKO: *n* = 9) and B16 (Flox/flox: *n* = 3, cKO: *n* = 4) tumors from Flox/flox or FROUNT-cKO mice. **c** Numbers of tumor-associated neutrophils, NK cells, CD4^+^, and CD8^+^ T cells in B16 tumors (*n* = 4). **d** Numbers of monocytes/macrophages and neutrophils in metastatic lungs (*n* = 6). **e** Confocal microscope images of macrophage accumulation (F4/80, red) around B16 lung metastatic nodules (green, broken outline). Three visual fields were averaged. **f** Pseudopodia protrusion of macrophages (F-actin staining, red), **g** monocyte (CD11b^+^Gr-1^−^Ly-6C^+^) chemotaxis and **h** effect of wortmannin (WM) (*n* = 3). Gating strategies to identify the immune cell populations are shown in Supplementary Fig. 10. *P < 0.05, ***P < 0.001 by a two-tailed, unpaired Student’s *t*-test. ns not significant. Error bars indicate s.e.m. Scale bars, 50 μm. N.D. not determined, MFI mean fluorescence intensity.

Next, we investigated the mechanisms responsible for FROUNT-mediated tumor progression. In FROUNT-GFP reporter (*Frount*-*gfp* knock-in) mice (Supplementary Fig. 5a–d), FROUNT expression was much higher in both Ly-6C^hi^ and Ly-6C^lo^ monocytes/macrophages than in other leukocyte subsets in tumor tissue, as well as in the bone marrow, which is a major source of tumor-associated macrophages^21^ (Fig. 3a and Supplementary Fig. 5e).

In *Frount*-deficient mice, we observed reduced accumulation of both Ly-6C^hi^ and Ly-6C^lo^ monocytes/macrophages in tumor tissue, as well as a decrease in the proportion of CD206^+^ M2 macrophages, which are associated with tumor progression^22^ (Fig. 3b), but no decrease in the other immune cell populations in the tumor (Fig. 3c), as well as the hematopoietic cell populations under the steady-state condition in the bone marrow and peripheral blood (Supplementary Fig. 6). In addition, we observed a selective reduction in the numbers of monocytes/macrophages in the lungs of metastasis model FROUNT-cKO mice (Fig. 3d), but not in steady-state lungs (Supplementary Fig. 6). Histological analysis of the local interaction of tumor cells and macrophages in the lungs revealed reduced accumulation of macrophages around metastatic nodules in *Frount*-deficient mice (Fig. 3e).

The expression of chemokines for CCR2 (*Ccl2* and *Ccl7*) or CCR5 (*Ccl3*, *Ccl4*, and *Ccl5*) in tumor-bearing lungs (Supplementary Fig. 7), chemoattractants for monocytes/macrophages, was unchanged in FROUNT-cKO mice. *Frount*-deficient macrophages exhibited comparable expression of CCR2 and CCR5, relative to control macrophages (Supplementary Fig. 4h). Rather, *Frount*-deficiency intrinsically impaired macrophage chemotactic responses to chemokine stimulation, as observed by defects in F-actin-rich lamellipodia-like pseudopodia protrusion (Fig. 3f) and impaired migration in response to CCL2 stimulation (Fig. 3g), which was inhibited by the PI3K inhibitor wortmannin (Fig. 3h).

### *Frount*-deficiency-induced changes in monocytes/macrophages

**Fig. 4:**
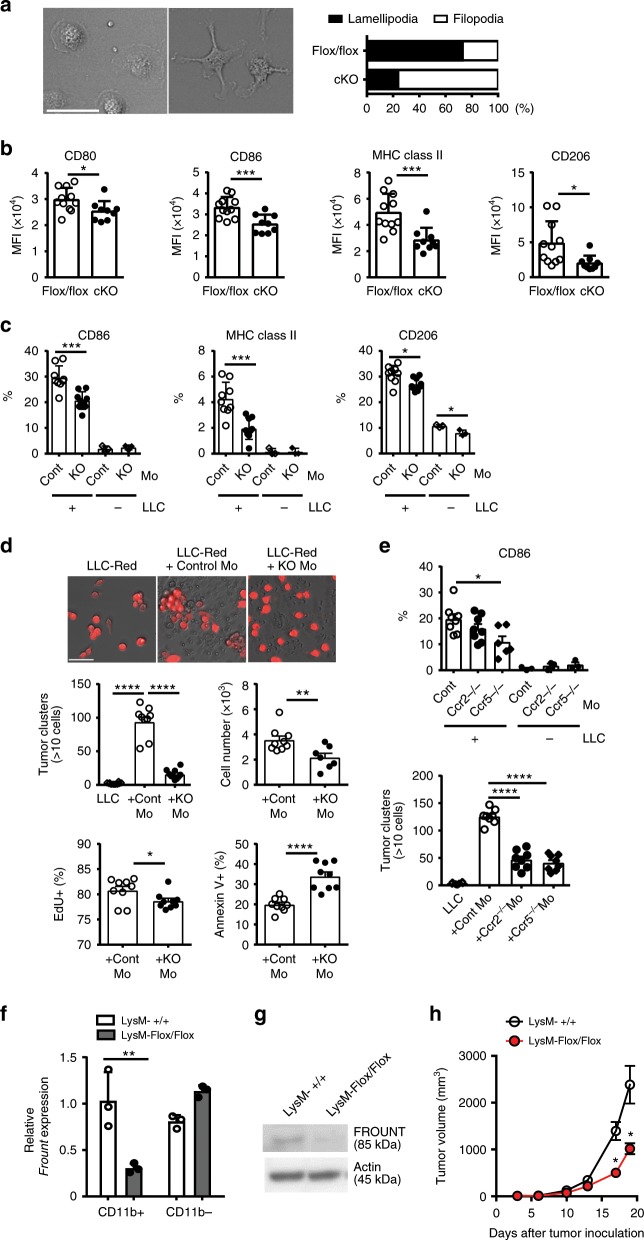
Morphological and functional changes in *Frount*-deficient monocytes /macrophages. **a** Morphology of macrophages isolated from tumor tissue of Flox/flox or FROUNT-cKO mice observed by differential interference-contrast microscopy (left panel) and ratios of lamellipodial to filopodial macrophages (based on analysis of >140 cells) (right panel). **b** In vivo expression of activation markers on macrophages in LLC-tumor tissue as measured by flow cytometry (Flox/flox: *n* = 11, cKO: *n* = 9). **c** In vitro expression of CD86, MHC class II and CD206 on monocytes from control (Cre^+^) or FROUNT-cKO (Cre^+^/Flox/flox) mice cultured with DsRed^+^ LLC tumor cells as measured by flow cytometry (*n* = 9). **d** Direct effect of macrophages on tumor cell growth in vitro. Representative image of DsRed^+^ LLC tumor cell clusters cultured alone or with monocyte-derived macrophages from control or FROUNT-cKO mice (upper panel) and enumeration of the clusters (>10 cells) per well (*n* = 9) (middle panel). Flow cytometry measurement of the number of DsRed^+^ tumor cells (middle panel), EdU^+^ proliferating tumor cells, and annexin V^+^ tumor cells (lower panel) (*n* = 9). **e** Effect of CCR2 or CCR5 deficiency on the upregulation of CD86 on monocytes, and tumor cell cluster formation in tumor-monocyte co-culture experiments (*n* = 8). **f–h** Myeloid cell-specific deletion of FROUNT in LysM-Cre mice with *Frount*-floxed allele (Flox/flox), and the wild-type allele (+/+) as a control. *Frount* mRNA expression in CD11b^+^ myeloid cells and CD11b^-^ nonmyeloid cells isolated from tumor tissue (**f**) (*n* = 3) and immunoblot analyses of FROUNT and pan-actin as a control in lysate of bone marrow-derived macrophages (**g**). **h** Myeloid cell-specific deletion of FROUNT inhibits tumor growth. Combined tumor-growth kinetics in LysM-Cre mice with the wild-type allele (+/+) or *Frount*-floxed allele (Flox/flox) subcutaneously inoculated with B16 tumors (*n* = 6 per group; representative of three independent experiments). *P < 0.05, **P < 0.01, ***P < 0.001, ****P < 0.0001 by two-tailed, unpaired Student’s *t*-test. Error bars indicate s.e.m. Scale bars, 50 μm.

We also observed altered morphology in ex vivo-cultured tumor-associated monocytes/macrophages from *Frount*-deficient mice, which had filopodia-like structures in contrast to the lamellipodia-like structures of control macrophages (Fig. 4a). These results are consistent with observations reported previously^14,18^. Tumor-associated macrophages in *Frount*-deficient mice also displayed lower expression of the activation markers CD80, CD86 and MHC class II, as well as M2 macrophage marker CD206, compared to those in control mice (Fig. 4b). On the other hand, when monocytes prepared from bone marrow cells were co-cultured with LLC tumor cells, we observed upregulation of the activation markers CD86, MHC class II and CD206 in control monocytes-derived macrophages, and this upregulation was impaired in *Frount*-deficient macrophages (Fig. 4c), suggesting that FROUNT mediates tumor-cell dependent activation of monocytes/macrophages. Macrophages are known to facilitate tumor cell growth and survival^23,24^. We investigated whether the altered phenotype of macrophages in *Frount-*deficient mice directly affects tumor cell growth in the co-culture experiments with monocytes. Control monocytes isolated from bone marrow induced vigorous growth of tumor cells, evident from the formation of abundant cell clusters, whereas *Frount-*deficient monocytes induce little change in the number of tumor cell clusters (Fig. 4d, upper panel). Consistent with these observations, the tumor cell number and EdU incorporation (an indicator of DNA synthesis) were higher in the control monocyte co-culture, while annexin V staining (an indicator of apoptosis) was higher in the *Frount*-deficient monocyte co-culture (Fig. 4d, the lower panel). These alterations in monocyte-tumor cell co-culture depend on chemokine receptors, CCR2 and CCR5, since co-culture of tumor cells with monocytes derived from *Ccr2-* or *Ccr5-*deficient mice resulted in impaired activation of the monocytes, as observed by decreased CD86 expression, especially in *Ccr5-*deficient monocytes, and decreased tumor cell cluster formation (Fig. 4e). These results suggest that FROUNT is involved in the ability of monocytes/macrophages to directly promote tumor cell growth and survival. In further support of macrophages playing a central role in FROUNT-mediated tumor progression, myeloid-specific deletion of *Frount* by crossing LysM-Cre transgenic mice with *Frount*-floxed mice resulted in decreased tumor growth (Fig. 4f–h). These findings suggest that *Frount*-deficiency results in morphological and functional changes in macrophages, leading to impaired tumor progression.

### Disulfiram inhibits chemokine-mediated FROUNT functions

**Fig. 5:**
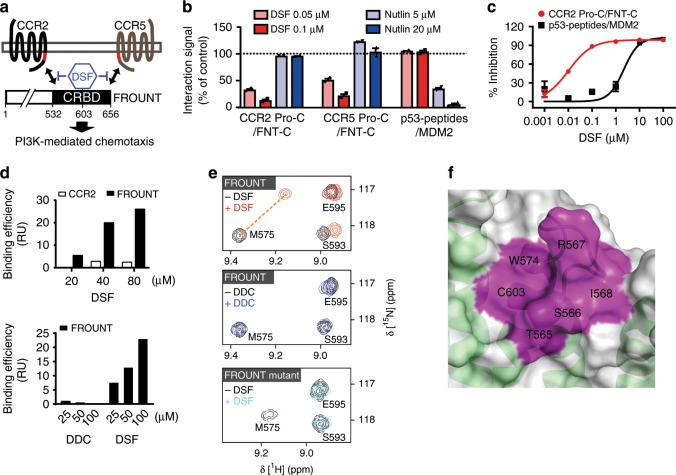
Disulfiram (DSF) binds FROUNT and inhibits its interaction with chemokine receptors. **a** DSF targets the interaction of CRBD (the chemokine receptor-binding domain) of FOUNT with CCR2 and CCR5 via a conserved binding region (shown in red). **b**, **c** HTRF interaction with DSF and the p53-MDM2 inhibitor, Nutlin (**b**) and titration assay (**c**). **d** Surface plasmon resonance assessment for DSF-binding signals to FROUNT versus CCR2 (upper panel) and FROUNT-binding signals of DSF versus its first metabolite diethyldithiocarbamate (DDC) (lower panel). **e** NMR titration analyses of the binding of DSF and DDC to FROUNT. Representative NMR peaks derived from FROUNT or its mutant (C603S) in ^1^H–^15^N HSQC spectra are shown. (Upper panel) The peaks before and after the 50 μM DSF titration to 50 μM FROUNT are shown in black and red, respectively. The largely perturbed M575 peaks (Δδ > 0.17 ppm) are connected by an orange dashed line. (Middle panel) The peaks before and after the 50 μM DDC titration to 50 μM FROUNT are shown in black and blue, respectively. (Lower panel) The peaks before and after the 50 μM DSF titration to 50 μM FROUNT mutant are shown in black and cyan, respectively. **f** Enlarged view of the putative DSF-binding site of FROUNT. Cartoon model of CRBD with a transparent surface and a map of the largely perturbed surface residues (Δδ > 0.17 ppm), labeled with their assignments and colored magenta. The other largely perturbed residues are buried inside the FROUNT structure. HTRF data in (**b**) and (**c**) show mean ± s.e.m.; duplicates from one of three independent experiments.

We next searched for FROUNT inhibitors that could be used to target tumor-promoting macrophages. Because FROUNT functions via interaction with a binding element in the membrane-proximal C-terminal region of the chemokine receptors CCR2 and CCR5^25^, we screened for compounds that disrupt the interaction with this region (Fig. 5a). Interestingly, one FROUNT inhibitor identified through multi-step screening of a library of 131,200 low-molecular weight compounds (Supplementary Fig. 8) was disulfiram (DSF), a known aldehyde dehydrogenase (ALDH) inhibitor and a clinically safe drug that has been in use for the treatment for alcoholism for over 60 years^26^. DSF specifically inhibited the interaction of FROUNT with both CCR2 and CCR5, which share the FROUNT-binding element (Fig. 5a, b). On the contrary, DSF did not affect the α-helix mediated p53-MDM2 interaction (which is similar but unrelated to the CCR2-FROUNT interactions), which was inhibited by the specific MDM2 inhibitor Nutlin^27^ (Fig. 5b). The IC_50_ of DSF for the FROUNT-CCR2 interaction was 42 nM, which is far more potent than the IC_50_ (>1000 nM) for the p53-MDM2 interaction (Fig. 5c). Unlike the effect of DSF on ALDH activity, where DSF metabolites have been shown to be more potent inhibitors than DSF itself^28^, DSF inhibited the FROUNT-CCR2 interaction more potently than its metabolites (Table 1).

**Table 1:** Inhibition properties of DSF and its metabolites.

IC_50_ values from inhibition experiments with disulfiram (DSF) and its metabolites, sodium diethyldithiocarbamate (DDC), DDC–copper complex (Cu(DDC)_2_), S-methyl-N,N diethylthiocarbamate sulfoxide (MeDTC sulfoxide), S-methyl-N,N-diethylthiocarbamate sulfone (MeDTC sulfone).

^§^Quoted from Mays et al., 1996^28^.

Surface plasmon resonance analysis showed that DSF directly binds to the FROUNT protein, but not to the FROUNT-binding region of CCR2, in a concentration-dependent manner (Fig. 5d, upper panel). In contrast, the first metabolite of DSF, diethyldithiocarbamate (DDC), did not bind to FROUNT (Fig. 5d, lower panel). Next, using NMR spectroscopy, we investigated the DSF-binding site on the chemokine receptor-binding domain (CRBD) of FROUNT (a.a. 532–656, Fig. 5a)^29^. For this analysis, L538E/P612S mutations were introduced to improve the protein solubility and spectral quality, without affecting the chemokine receptor-binding activity^30^. Recently, we determined the three-dimensional structure of CRBD by NMR, in which the region of a.a. 534–648 is well structured (PDB ID: 6L5C). DSF titration to CRBD induced specific chemical shift perturbations in CRBD (Fig. 5e, upper panel) and 13 residues (a.a. 564–569, 574, 575, 578, 579, 600, 603, and 605) were largely perturbed (Δδ > 0.17 ppm); however, there was no significant perturbation upon DDC titration (Fig. 5e, middle panel). The perturbed amino acids form a pocket that appears to accommodate DSF in the structural domain of CRBD (Fig. 5f). We synthesized a CRBD protein with a point mutation (C603S) in one of the perturbed amino acids and performed an NMR titration analysis of the DSF-binding activity (Fig. 5e, lower panel), since C603 is located in the putative DSF-binding pocket (Fig. 5f). No significant binding of DSF to the C603S FROUNT was observed, indicating that the mutant does not retain the DSF-binding activity and that the proposed pocket is the DSF-binding site of FROUNT. These experimental results showed that DSF, but not DDC, binds to CRBD of FROUNT, and therefore, DSF itself, but not its metabolites, inhibits the FROUNT–chemokine-receptor interactions.

**Fig. 6:**
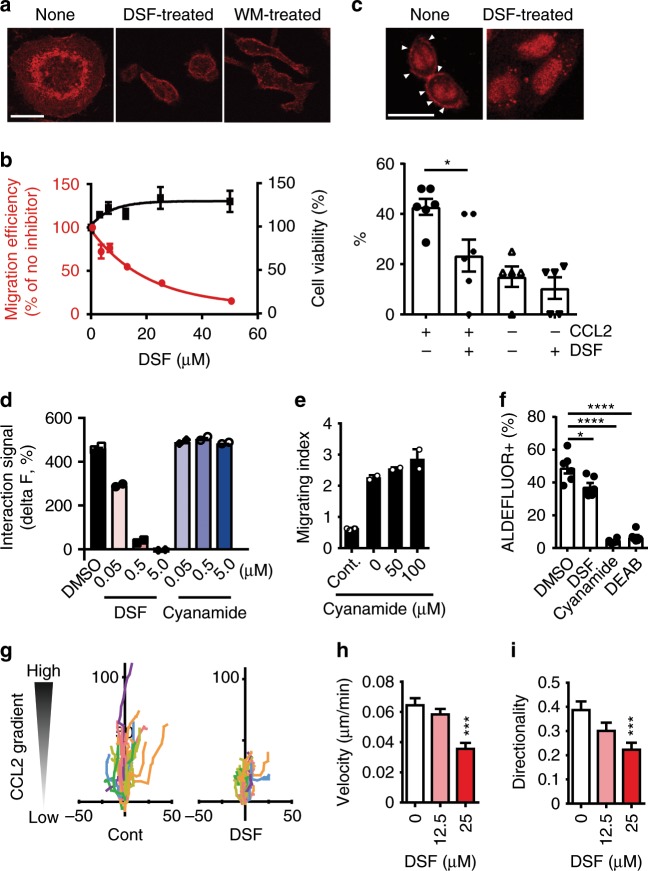
Disulfiram (DSF) is a potent inhibitor of chemokine-mediated FROUNT functions. **a** Effect of DSF (25 µM) on CCL2-induced pseudopodia protrusion compared with DMSO control and wortmannin (WM, 25 µM). **b** Effect of DSF on chemotaxis (red line), and cell viability (black line) (mean ± s.e.m., *n* *=* 3). **c** Translocation of PH-Akt-tHcRed to the plasma membrane as an indicator of PI3K activation in CCR2 + Chinese hamster ovary cells expressing PH-Akt-tHcRed, in the presence or absence of DSF upon stimulation with CCL2. Representative confocal microscopic images of cells stimulated with CCL2 (left) and percentage of cells in which PH-Akt-tHcRed was translocated to the membrane. Arrowheads indicate sites of membrane translocation of PH-Akt-tHcRed. More than five visual fields were averaged. **d, e** The ALDH inhibitor cyanamide did not block FROUNT-CCR2 interactions or chemotaxis (mean ± s.e.m., duplicates from one of three independent experiments.). **f** ALDH inhibition activity of DSF and cyanamide evaluated by ALDEFLUOR assay (mean ± s.e.m., *n* *=* 6). **g**–**i** TAXIScan assay^57^ for migration of THP-1 cells treated with DMSO (control) or DSF (50 μM) towards CCL2. **g** cell tracks, **h** velocity, and **i** directionality. Plots indicate means of 28 cell tracks ± s.e.m. *P < 0.05, ***P < 0.001, ****P < 0.0001 by two-tailed unpaired Student’s *t*-test as compared to control. Scale bars, 20 μm.

As expected, the blockade of the FROUNT-CCR2 interaction by DSF inhibited CCL2-induced chemotactic response with pseudopodia protrusion (Fig. 6a) and cellular chemotaxis without any cytotoxicity at the concentrations tested (Fig. 6b). These chemotactic responses were PI3K-dependent because of inhibition by the PI3K inhibitor, wortmannin (Fig. 6a). To further assess the involvement of DSF in PI3K activation, we evaluated translocation of the pleckstrin homology domain of Akt (PH-Akt) from the cytosol to the plasma membrane, which is dependent on PI3K activation. DSF treatment significantly impaired membrane localization of PH-Akt upon CCL2 stimulation (Fig. 6c). In contrast, the ALDH inhibitor cyanamide^31^ inhibited neither FROUNT-CCR2 interactions (Fig. 6d) nor chemotaxis (Fig. 6e), although it did inhibit ALDH activity more potently than DSF, as assessed by ALDEFLUOR assay (Fig. 6f), excluding the possibility of an ALDH-dependent mechanism underlying the effects of DSF in our experiments. We also observed specific uptake of ^11^C-labeled DSF in FROUNT-overexpressing cells (manuscript in preparation). Lastly, in the chemotaxis imaging of human monocytic THP-1 cells, DSF impaired chemotaxis in terms of both velocity and directionality (Fig. 6g–i), consistent with the effects of FROUNT knockdown reported previously^15^. These results indicate that DSF inhibits FROUNT-dependent, PI3K-mediated cellular chemotactic responses.

### Disulfiram blocks tumor progression via macrophage regulation

**Fig. 7:**
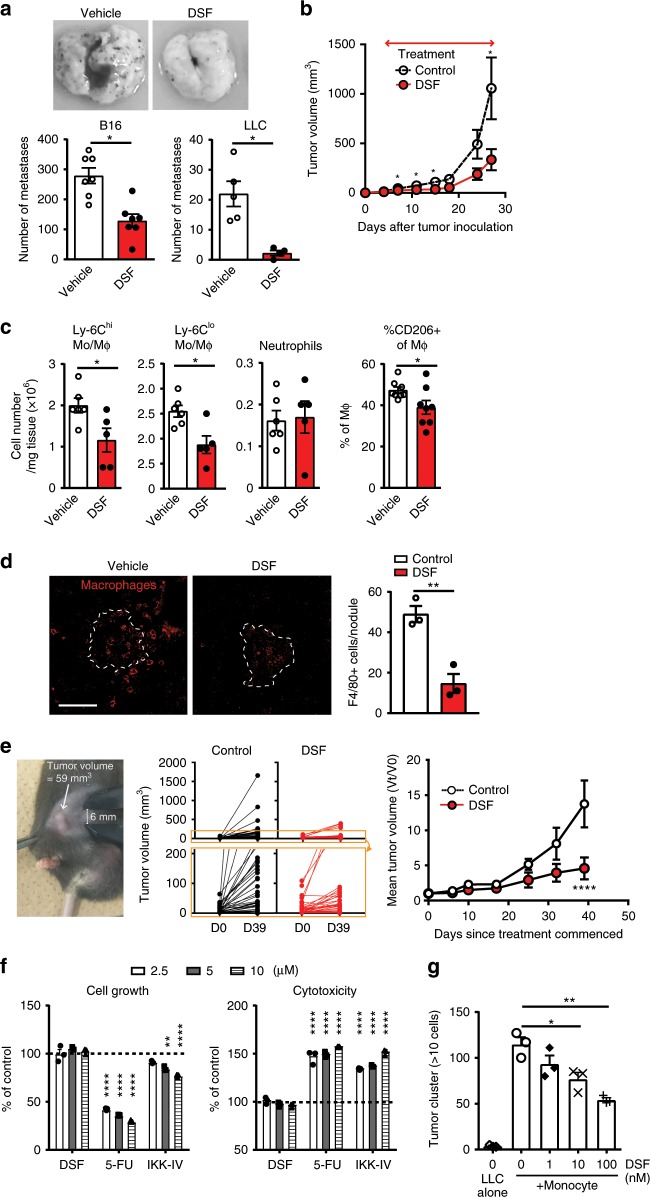
Disulfiram (DSF) inhibits tumor-growth and targets tumor-promoting macrophages. **a** Effect of DSF on B16 and LLC tumor metastasis in C57BL/6 mice (*n* = 7 for B16 and *n* = 5 for LLC). **b** Effect of oral DSF (from 4 days after tumor inoculation) on LLC subcutaneous tumor growth (*n* = 10). **c** Numbers of Ly-6C^hi^ and Ly-6C^lo^ monocytes/macrophages and neutrophils in tumors, and proportion of CD206^+^ M2-type cells among tumor-associated macrophages (CD45^+^CD11b^+^CD24^−^Ly-6G^−^ cells in leukocyte gate) on day 20 after tumor inoculation. (*n* *=* 5). **d** Accumulation of macrophages (F4/80, red) around metastatic lung nodules (broken outline) (day 12 after B16 tumor cell injection). Three visual fields were averaged. **e** Effect of DSF on tumor growth in the MMTV-PyVT spontaneous mammary tumor model. Representative image of tumor at treatment start (day0) (left), individual tumor sizes at day0 and 39 (middle) and combined growth rate curves of each tumor (4 mice each group, 10 mammary tumor sites per mice). **f** Effects of 48-h in vitro culture in the presence of DSF, the cytotoxic anti-cancer drug 5FU, and the NF-κB inhibitor IKK-IV on LLC tumor cell growth (left panel, WST-1 assay, s.e.m. for the control = 1.298) and toxicity (right panel, LDH cytotoxicity assay) (assayed in triplicate, representative of three independent experiments, s.e.m. for the control = 0.5201). **g** Direct action of DSF on monocyte-derived macrophages function to influences tumor cell growth in vitro. The experiment was repeated three times. *P < 0.05, **P < 0.01, ****P < 0.0001 by two-tailed unpaired Student’s *t*-test. Error bars indicate s.e.m. Scale bars, 50 μm.

We next examined the effect of DSF on tumor growth in vivo. DSF administration significantly reduced lung metastatic nodule formation following intravenous injection of B16 or LLC tumor cells (Fig. 7a) and inhibited subcutaneous tumor growth (Fig. 7b) at a dose equivalent to the clinical dosage used to treat alcoholism. DSF treatment also reduced the number of tumor-associated monocytes/macrophages but not neutrophils, with a decreased ratio of M2 macrophages (Fig. 7c), and diminished macrophage accumulation around tumor metastatic nodules (Fig. 7d). To further confirm the antitumor effect of DSF, we tested the MMTV-PyVT spontaneous mammary tumor model. DSF treatment impaired the growth of established tumors (Fig. 7e), suggesting the therapeutic potential of DSF for cancer treatment.

DSF-mediated direct tumor-killing activity has been reported in certain tumor cell lines and is known to be associated with mechanisms such as the NF-κB pathway^32,33^ and NPL4^34^. However, in the LLC and B16 tumor cells used in our experiments, DSF had no effect on in vitro growth or viability, whereas both the cytotoxic drug 5FU and the NF-κB inhibitor IKK-IV affected LLC and B16 cell growth and viability (Fig. 7f and Supplementary Fig. 9a). Furthermore, in consistent with that from *Frount*-deficient monocytes/macrophages (Fig. 4d), the tumor cell growth induced by co-culture with monocytes was inhibited in the presence of nano-molar concentrations of DSF (Fig. 7g). Taken together, our results demonstrate that DSF inhibits growth of the tumor, which is insensitive to the direct tumor-killing effect of DSF, suggesting the mechanism being mediated via its FROUNT-targeted macrophages regulation.

### Synergism between disulfiram and immune-checkpoint blockade

**Fig. 8:**
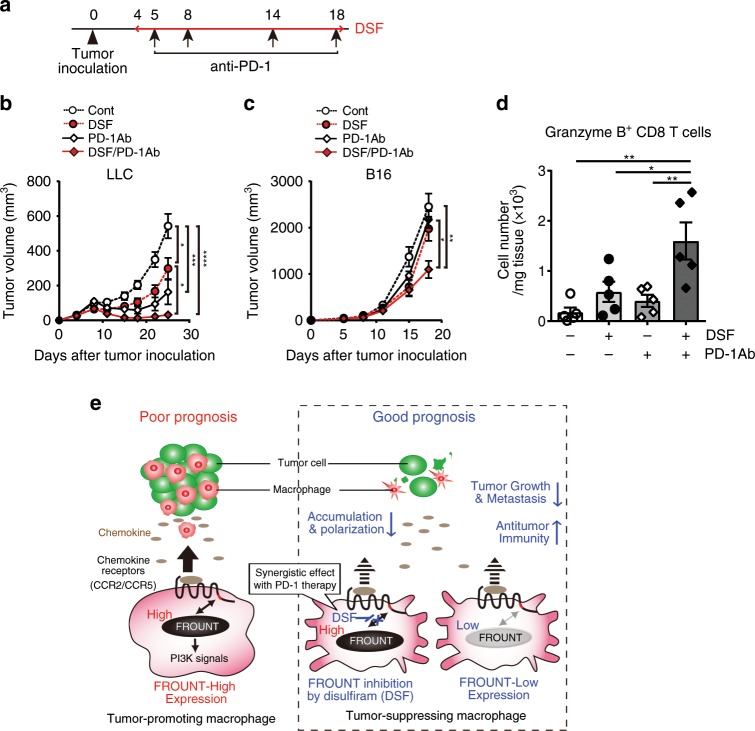
Synergism between disulfiram (DSF) and immune-checkpoint blockade. **a**–**d** Effects of combination treatment with DSF and immune-checkpoint inhibitor anti-PD-1 antibody on subcutaneous LLC (**b**) and B16 (**c**) tumor growth (*n* = 9). **d** The number of Granzyme B^+^ CD8^+^ T cells at day 20 post inoculation, detected by flow cytometry (*n* = 5). **e** Scheme illustrating the mechanisms by which cytoplasmic protein FROUNT modulates tumor-associated macrophages. The experiment was repeated three times. *P < 0.05, **P < 0.01, ***P < 0.001, ****P < 0.0001 by one-way ANOVA with Tukey’s multiple comparison test. Error bars indicate s.e.m.

Synergistic effects of PI3Kγ inhibition and immune-checkpoint blockade via myeloid cell-dependent mechanisms have been reported in mouse tumor models^35,36^. We co-treated mice with DSF and an antibody against the immune-checkpoint PD-1 to determine whether similar synergy might be observed with DSF (Fig. 8a–d). In the LLC (Fig. 8b and Supplementary Fig. 9b) and B16 subcutaneous models (Fig. 8c and Supplementary Fig. 9c), significant additive and synergistic effects, respectively, were observed compared to single-agent therapy. Combined DSF and PD-1 antibody treatment markedly increased the number of granzyme B-positive CD8^+^ T cells in the tumor (Fig. 8d) compared to monotherapy, suggesting the cytotoxic T-cell (CTL)-dependent mechanism for the combination effect and that macrophage regulation by the FROUNT inhibitor DSF might increase the responsiveness to immune-checkpoint therapy.

## Discussion

There is considerable interest in cancer therapies that target molecules mediating macrophage activity, such as chemokines^11–13,16,17^, PI3Kγ^35,36^, and CSF1^37^. In this study, we revealed a critical role of intracellular chemokine signal regulator FROUNT in tumor-promoting macrophages (Fig. 8e). Both knockout and small compound-mediated functional inhibition of FROUNT impairs tumor progression in mice. In addition to the reduction of macrophage accumulation in tumor sites as expected from FROUNT function, we also found impaired activation of macrophages due to *Frount* deficiency, accompanied by reduced expression of M1-like macrophage markers, such as CD86 and MHC class II, and impaired expression of M2 macrophage markers. These paradoxical properties of *Frount*-deficient macrophages might affect the direct tumor-promoting properties of macrophages, especially at the early stage when they encounter tumor cells as observed in the macrophage-tumor cell co-culture experiments (Fig. 4c–e). However, it should be further investigated for establishing a mechanistic link between this phenotypic alteration of macrophages and antitumor effect.

The chemokine receptors CCR2 and CCR5 are major chemotactic regulators of tumor-promoting macrophages at the tumor site, and blockade of these receptors impair tumor progression in experimental animal models^10–13,16,17,38,39^. However, these findings have not yet been translated into effective clinical therapies, possibly due to species-specific differences in chemokine receptors or due to redundancy in chemokine signaling. FROUNT promotes chemotactic signaling via CCR2 and CCR5 by binding to a conserved intracellular region of these receptors^14,15,25,40^. Since FROUNT is a molecule involved in signal amplification, its inhibition is expected to have effects different from that of the inhibition of chemokine or chemokine-receptor blockade, as evidenced by the minimal effect on steady-state monocyte population in *Frount*-deficient mice (Supplementary Fig. 6). This study also showed comparable expression of FROUNT-binding receptors CCR2 and CCR5 in TAMs. We had previously reported the functional involvement of FROUNT in chemokine-receptor signaling, even at equivalent expression of the receptor^25^. Because FROUNT and the FROUNT-binding region in CCR2/CCR5 are highly conserved across species, results in mice are likely to be applicable in humans, and FROUNT represents a promising therapeutic target via regulating both CCR2/CCR5 signaling.

FROUNT is not only highly expressed in macrophages but also ubiquitously expressed at lower levels in a broad range of cell types. The significant reduction in tumor cell growth following myeloid cell-specific deletion of FROUNT in LysM-Cre Flox/Flox mice (Fig. 4f–h) suggest that FROUNT in monocytes/macrophages is likely to be an important target for the antitumor effects of DSF. Selective reduction of the number of monocytes/macrophages in tumors, observed in *Frount*-deficient mice (Fig. 3b–d), also suggests that monocytes/macrophage FROUNT is a major target for the antitumor effects of DSF. However, since the impact of *Frount*-deficiency on tumor growth was less prominent in myeloid-specific *Frount*-deficient mice than in whole knockout mice, the potential roles of FROUNT expression in other cell types (including nonmyeloid cells) during tumor development cannot be excluded, and should be investigated in future studies.

DSF potently inhibits FROUNT by directly binding to the FROUNT and interfering with FROUNT-chemokine-receptor interactions. This is the first report of FROUNT inhibitor directly binding to FROUNT, and its therapeutic effect is demonstrated by using an animal disease model, although there has been a recent report on an inhibitor of FROUNT-chemokine-receptor interactions assessed using cultured cells^41^. The direct tumoricidal activity of DSF has been known for some time, which was mediated via an ALDH-dependent mechanism in the context of cancer stem cells^42^ or, via the NF-κB pathway^32,33^. These observations have led to several clinical trials, including those on glioblastoma (NCT02678975), melanoma (NCT00256230, NCT00571116), lung cancer (NCT00312819), and prostate cancer (NCT01118741). Recently, a large-scale epidemiological study showed significant clinical impact of continuous DSF treatment on cancer-related mortality^34^. Importantly, in our experiments, DSF did not display any direct tumoricidal activity against the B16 or LLC tumor cell lines, suggesting that, in our tumor models, the antitumor effects of DSF were due to the regulation of the tumor microenvironment, although further comprehensive investigations are needed to elucidate specific factor involved in FROUNT-mediated antitumor responses. Furthermore, the tumoricidal effects of DSF demonstrated in previous reports have been attributed to its metabolites^32,34,43,44^, whereas, the FROUNT-targeted effects described in this study were mediated more potently by DSF itself than by the metabolite (Table 1). Our structural analysis suggested that a hydrophobic property of the entire chemical structure of DSF is required to interact with FROUNT, while the DDC form is insufficient to bind to the binding pocket of FROUNT since the hydrophobic property is reduced and an acidic property of the formed dithiocarboxy group (CSS-) is added, although the physiological contribution of the DSF-binding C603 residue to inhibition activity of DSF, as well as to the function of FROUNT protein should be further addressed in future work. Accordingly, our results reveal a previously unknown aspect of the antitumor effect of DSF. Therefore, we intend to further investigate these FROUNT-targeted effect using other FROUNT-specific inhibitors, that are under development in our research group. To define the clinical significance of FROUNT-targeted therapy, it will also be necessary to determine the correlation between responsiveness to DSF treatment and FROUNT expression in cancer patients.

In clinical and experimental studies^36,45^, greater macrophage infiltration is observed in nonresponders to immune-checkpoint antibody therapy, raising the possibility that macrophages reduce the efficacy of immune-checkpoint blockade therapy, and that macrophage-targeted combination therapies may improve response rates of immune-checkpoint antibody therapy. Interestingly, CTL activation similar to the increase in granzyme B^+^ CD8 T cells that we observed following combination treatment of an immune-checkpoint antibody with DSF has also been reported with other macrophage-targeted therapeutic approaches^35,36^. The identification of DSF, a clinically safe and inexpensive drug, as a FROUNT inhibitor that has synergistic antitumor effects when combined with immune-checkpoint therapy suggests that modulation of chemokine signaling by targeting FROUNT represents a promising and readily realizable strategy for macrophage-targeted cancer therapy. Thus, our findings of the chemokine-receptor-associating molecule FROUNT as a new class of target to control tumor-promoting macrophages, and the inhibitory activity of the old drug disulfiram against FROUNT function, provides an effective therapeutic option in cancer treatment, focusing on intracellular chemokine signaling molecules. Based on these observations, we have started to investigate disulfiram in combination with anti-PD-1 antibody in patients with gastric cancer (jRCTs031180183)^46^.

## Methods

### Study subjects

This study consisted of 40 female patients with lung adenocarcinoma greater than stage II treated in the Department of Thoracic Disease, Chiba Cancer Center from 1997 to 2004^47^. Patients provided written informed consent to participate, then underwent complete resection of the lobe or segment in which the tumor resided. After resection, the surgical samples were immediately snap-frozen in liquid nitrogen and stored at −80 °C until analysis. For validation, samples from 31 additional patients were collected based on the same criteria from the same center. For histological examination, samples were fixed in 10% formalin, embedded in paraffin-embedded blocks, then cut into 4-μm-thick sections. For routine histological studies, sections were stained with hematoxylin and eosin. For FROUNT detection, sections were stained with rabbit anti-FROUNT polyclonal antibodies raised in our laboratory^14^ followed by biotin-conjugated anti-rabbit IgG (BD Pharmingen) and horseradish peroxidase streptavidin (BD Pharmingen), with subsequent nuclear staining. To detect tumor-associated macrophages^48^, paraffin sections were treated by heating them in a pressure cooker (DAKO) with 1 mmol L^−1^ EDTA buffer (pH 8.0). Then, sections were stained with an anti-CD68 antibody (clone PG^−^M1, 1 µg mL^−1^, DAKO), followed by a horseradish peroxidase-labeled anti-mouse IgG antibody (Nichirei). 3,3-diaminobenzidine was used to visualize positive signals. For double-immunostaining, sections were retreated with EDTA buffer in a pressure cooker and then reacted with an anti-FROUNT polyclonal antibody^14^ (rabbit polyclonal, 1 µg mL^−1^). They were then treated with a horseradish peroxidase-labeled anti-rabbit IgG antibody (Nichirei), and the positive signal was visualized using HistoGreen substrate (#AYS-E109; Linaris, Dossenheim, Germany) as the 2nd step.

Pathological staging was determined using the current tumor node metastasis classification system (International Association for the Study of Lung Cancer). The histological type and the grade of cell differentiation in these patients were determined using the pathological diagnosis by two pathologists at the Chiba Cancer Center of Pathology, and then confirmed according to the World Health Organization Classification System 2004 by a pathologist specializing in lung pathology. The study was approved by the institutional review boards/ethics committees of the Chiba Cancer Center^47^, Kumamoto University Hospital Review Board, the Research Ethics Committee of the Faculty of Medicine, University of Tokyo, and Ethics Committee of Tokyo University of Science.

### Survival analysis

To investigate the relationship between *FROUNT* expression and the prognosis of patients with lung adenocarcinoma, total RNA was isolated from surgical specimens and expression of *FROUNT* and the internal control *GAPDH* was measured using quantitative real-time RT-PCR as described below. Overall survival and recurrence-free survival were defined as the time from surgery to death and to the first evidence of recurrence, respectively. Patients lost to follow-up were censored. For the Cox proportional hazards model, we used log2 *FROUNT* expression as a variable, defined as the log-transformed relative ratio of *FROUNT* (*NUP85*) standardized by reference (*GAPDH*) mRNA levels, and calculated the hazard ratios and 95% confidence intervals. *FROUNT* expression was a significant risk factor for recurrence and survival, with a 2-fold increase in *FROUNT* expression being correlated to a 2.0-fold increased risk of recurrence (*P* = 0.0037) and a 1.9-fold increased risk of patient survival (*P* = 0.015). Based on the relative *FROUNT* mRNA median values for their lung specimens, patients with lung cancer were divided into two groups: *FROUNT*-high and FROUNT-low groups. Survival was compared using Kaplan-Meier analysis with the log-rank test. Correlation between FROUNT mRNA expression and expression of the myeloid cell-related immunosuppressive markers CD204, PD-L1 and PD-L2 were measured. The primers used were as follows: *FROUNT*, forward 5′-GCTGCTAAAGATCCAGCCAAT-3′ and reverse 5′-GATGAGCTCCATTGCTGACA-3′; *GAPDH*, forward 5′-GAAGGTGAAGGTCGGAGTC-3′ and reverse 5′-GAAGATGGTGATGGGATTTC-3′; *MSR-1_CD204*, forward 5′-TCGAGGACTCCCAGGATATG-3′ and reverse 5′-TGTGTTTCCACTCCCCTTTT-3′; *PD-L1*, forward 5′-GCATGGAGAGGAAGACCTGA-3′ and reverse 5′- TTGTAGTCGGCACCACCATA-3′; *PD-L2*, forward 5′-CAGCAATGTGACCCTGGAAT-3′ and reverse 5′-GGACTTGAGGTATGTGGAACG-3′. These survival analyses were conducted with R software (www.r-project.org). For mutation detection of the *EGFR*, *KRAS*, and *p53* genes, each exon of the *EGFR* gene (exons 18–21), *KRAS* gene (exons 2) and *TP53* gene (exons 4–8) was amplified by PCR^47^. The primers used were as follows: *EGFR ex.18*, forward 5′-CCGTGTCCTGGCACCCAAGC-3′ and reverse 5′-CCCAAACACTCAGTGAAACAAAGAGTAAAG-3′; *EGFR ex.19*, forward 5′-CCTTAGGTGCGGCTCCACAGC-3′ and reverse 5′-GTGAACATTTAGGATGTGGAGATGAGCAG-3′; *EGFR ex.20*, forward 5′-TAAACGTCCCTGTGCTAGGTCTTTTGC-3′ and reverse 5′-CATGCAGATGGGACAGGCACTGA-3′; *EGFR ex.21*, forward 5′- CAGCCATAAGTCCTCGACGTGG-3′ and reverse 5′-CATCCTCCCCTGCATGTGTTAAAC-3′; *KRAS ex.2*, forward 5′-ACGATACACGTCTGCAGTCAACTGGAAT-3′ and reverse 5′-CCCTGACATACTCCCAAGGAAAGTAAAG-3′; *TP53 ex.4*, forward 5′-CAGACTTCCTGAAAACAACGTTCTGGTAAG-3′ and reverse 5′-TTGGGACAGGAGTCAGAGATCACACATT-3′; *TP53 ex.5*, forward 5′-TCTCTCTAGCTCGCTAGTGGGTTGC-3′ and reverse 5′-TACTCCACACGCAAATTTCCTTCCACTC-3′; *TP53 ex.6*, forward 5′-TCACAGCACATGACGGAGGTTGTGAG-3′ and reverse 5′-CACATCTCATGGGGTTATAGGGAGGT-3′; *TP53 ex.7*, forward 5′-TGGTGCTGGGCACCTGTAGTCC-3′ and reverse 5′-AGAAAACTGAGTGGGAGCAGTAAGGAGA-3′; *TP53 ex.8*, forward 5′-CCACCTACCTGGAGCTGGAGCTTA-3′ and reverse 5′-GCTGGGGAGAGGAGCTGGTGTT-3′;

### Human macrophage culture and immunocytostaining

Peripheral blood mononuclear cells were obtained from healthy volunteer donors who had all provided written informed consent for the use of their cells in accordance with the study protocols approved by the Kumamoto University Hospital Review Board (#1169).

Monocytes were isolated using RosettSep cocktail (StemCell Tech., Vancouver, Canada). Then monocytes were cultured in 2% human serum, 1 ng mL^−1^ granulocyte macrophage‐colony stimulating factor (WAKO), and 50 ng mL^−1^ macrophage‐colony stimulating factor (WAKO) for 7 days to induce macrophage differentiation. For immunocytostaining, cells were treated by cold acetone and then reacted with anti-CD68 antibody (PM-1K, 1 µg mL^−1^, Transgenic, Kumamoto) and anti-FROUNT antibody (#19370-1-AP, 1 µg mL^−1^, Peprotech). Then they were reacted by Alexa Fluor 488- labeled anti-mouse IgG antibody and Alexa Fluor 546- labeled anti-rabbit IgG antibody (Invitrogen).

### RNA-Seq and data processing

RNA-seq samples were acquired using Illumina TruSeq RNA Sample Preparation Kit (Illumina, Inc., San Diego, CA, USA) by purifying the mRNA poly-A tails with poly-T oligonucleotide-attached magnetic beads followed by thermal fragmentation and cDNA reverse transcription with recombinant reverse transcriptase and random primers. cDNA synthesis was performed using DNA polymerase I and RNase H. Upon end repair, single A bases were appended followed by adapter ligation before purification and amplification with PCR to create a cDNA library for sequencing (Macrogen, Seoul, South Korea). Read alignment against hg19 human reference genome was achieved using TopHat v2.0.11^49^ and Bowtie 2.1.0^50^, followed by Cufflinks v2.1.1^51^ to process alignments into read counts in units of fragments per kilobase of exon per million fragments mapped (FPKM)^52^.

### Analysis of human transcriptome databases

Datasets were selected from PRECOG and PrognoScan using the FDR (Benjamini & Hochberg) method where *q* < 0.05 (the *q*-value is a measure of the strength of an observed statistic with respect to FDR^20^). For each dataset, the Cox hazard model (p[Cox]) was used for evaluating the association of FROUNT expression with survival. Patients were divided into two groups based on median FROUNT expression, and log-rank tests (p[LogRank] were used to compare the survival between *FROUNT*-high (red line) and -low groups (blue line). HumanProteinAtlas was also used to obtain survival analysis for FROUNT (NUP85) using median separation.

### Animal care and generation of FROUNT genetically modified mice

Homozygous *Frount* (*Nup85*)-null mice, *Frount-*floxed (Flox/flox) mice, and *Frount*-*gfp* knock-in (*Frount*-flox/*gfp*, FROUNT-GFP reporter) mice were generated in our laboratory using C57BL/6 embryonic stem cells. Homozygous *Frount*-null mice died at the embryonic stage. To generate tamoxifen-inducible *Frount*-conditional knockout (FROUNT-cKO) mice, *Frount*-floxed or *Frount*-flox/*gfp* mice were crossed with CAG-Cre/ER transgenic mice (Jackson Laboratories, #004682) in which the expression of tamoxifen-inducible Cre/ER recombinase was broadly driven by the cytomegalovirus early enhancer element and β-actin promoter. Age-matched FROUNT-cKO mice and littermate *Frount*-floxed mice were fed with the CE-2 diet (CLEA Japan Inc.) containing tamoxifen citrate (Wako) at a concentration of 0.4 mg per 1 g CE-2 diet from 6 or 14 days prior to the experiments to induce targeted recombination. In some experiments, myeloid cell-specific FROUNT-cKO mice were generated by crossing *Frount*-floxed mice with LysM-Cre Tg (Jackson Laboratories, #004781) and LysM-Cre Tg mice without the floxed allele were used as controls. B6.FVB-Tg (MMTV-PyVT) 634Mul/LellJ were purchased from Jackson laboratory (#022974). Recombination was confirmed by Southern blotting, western blotting, and PCR analysis. To confirm myeloid cell-specific deletion of FROUNT in LysM-Cre Flox/Flox mice, CD11b^+^ myeloid cells and CD11b^−^ nonmyeloid cells were isolated from tumor tissue by anti-CD11b MACS beads and autoMACS (Miltenyi Biotec). The primers for PCR used in Supplementary Fig. 4 were:

FROUNT-#1 (forward TCAGCCAGTGACCTTGGAAC, reverse GTACAGCTGTACTGGTTGTAC); FROUNT-#2 (forward AGAGTCCTATGTAGGTGGGAGGTT, reverse GAACATCTATGGGATCACTGTCAAT); Cre (forward TTCCATGGAGCGAACGACGAGACC, reverse AGGTAGTTATTCGGATCATCAGCTA); Knock-in (forward CGTAAACGGCCACAAGTTCAG, reverse TGTGATCGCGCTTCTCGTT); 5′-probe (forward ACTATAGGCTGTGATGAGCTGACAT, reverse TGCAAATGTGTTGTCTTGTAAGTGT); 3′-probe (forward GTCTCTTCAACATATGGTAGGCATC, reverse CCAGTACCAACTAATGGCCTCTAC); Neomycin (forward GAACAAGATGGATTGCACGCAGGTTCTCCG, reverse CGCCAAGCTCTTCAGCAATA). *Ccr2*-knockout mice on the C57BL/6 background were purchased from Taconic Biosciences and *Ccr5* knockout mice on the C57BL/6 background were generated in our laboratory^53^. Wild-type C57BL/6 mice were purchased from Japan SLC. All animal studies were performed in accordance with the guidelines of the Animal Care and Use Committee of the University of Tokyo and Animal Care and Use Committee of Tokyo University of Science.

### Cell lines

LLC, B16F10 (B16), THP-1 cells, and CHO cells were obtained from the American Type Culture Collection. For visualization of tumor cells, B16-DsRed and LLC-DsRed cell lines were established by retroviral transduction; >95% cells were positive for DsRed fluorescence.

### Tumor models

For evaluating subcutaneous tumor growth, LLC or B16 cells (5 × 10^5^) in 50 μl PBS were inoculated subcutaneously into the right flanks of mice. Mice were euthanized when their tumor volumes reached 4000 mm^3^. Tumor sizes were measured twice a week using calipers. Tumor volume was calculated using the following formula: Volume = (width)^2^ × length/2. For the lung metastasis model, 1 × 10^6^ LLC or B16 cells in 200 μl PBS were injected intravenously into mice. Mice were euthanized at day 10 after tumor injection for the LLC model and day 5–9 for the B16 model, and the lungs were isolated after perfusion with PBS via the left ventricle. Visible lung metastases were counted in the left lobe. The diameters of metastatic nodules in the lung lobe image were measured using ImageJ software. To test the antitumor effects of disulfiram (DSF) (Tokyo Chemical Industry) in lung metastasis models, DSF was administered intraperitoneally at a dose of 40 mg kg^−1^ daily from one day prior to tumor injection. To test the antitumor effects in a subcutaneous tumor-growth model, mice were grouped into control and treatment groups based on their tumor size and fed daily with DSF in combination with a CE-2 powder diet (0.8 mg DSF/1 g CE-2) containing 5% sucrose (Wako), commencing 4 days (LLC) and 5 days (B16) after tumor inoculation or control diet without DSF. For evaluation of antitumor effect of DSF in spontaneous mammary tumor model, female MMTV-PyVT transgenic mice were randomly divided into two groups and treated with DSF beginning at the age of 17 weeks and tumor sizes in mammary fat pads were measured twice a week using calipers. Anti-PD-1 antibody (clone J43, BioXcell) was injected intraperitoneally at a dose of 200 μg per mouse on day 5, 8, 14, and 18 after tumor inoculation.

### Chemotaxis inhibition assay

THP-1 cells were counted, resuspended in chemotaxis buffer [0.1% BSA, 10 mM HEPES, 100 U mL^−1^ penicillin-streptomycin (Life Technologies), and L-glutamic acid (Sigma–Aldrich) in RPMI medium without phenol red (Life Technologies)] and pre-incubated with the inhibitors DSF or cyanamide (Sigma–Aldrich) before chemotaxis assays. Chemotaxis was measured in a 96-well chemoTX chemotaxis chamber with a polycarbonate filter (5-μm pore size) (Neuro probe). Human CCL2 (R&D Systems) was added to the lower chamber of the plate and the cells were added to the upper chamber. After incubation at 37 °C and 5% CO_2_ for 90 min, the filter was removed and the number of migrated cells in the lower chamber was counted using a cell counting kit F (Dojindo). Data are expressed as migration efficiency (percentage of the maximal migration).

### Flow cytometry-based chemotaxis assay

Bone marrow cells were mechanically flushed from the femurs and tibias of *Frount*-floxed mice and FROUNT-cKO mice. After incubation in ammonium chloride to lyse red blood cells, nucleated cells were incubated with the following antibodies: PE-conjugated Gr-1 (clone RB6-8C5, dilution 1:400, Cat 553128), Pacific blue-conjugated Ly-6C (clone RB6-8C5, dilution 1:400, Cat 128014), and allophycocyanin-conjugated CD11b (clone M1/70, dilution 1:400, Cat 101212). Cells were washed and adjusted to 2 × 10^7^ cells mL^−1^ in chemotaxis buffer. Cells were pre-incubated with 20 μM wortmannin before the assay as necessary. The chemokines murine CCL2 (Peprotech) were added to the lower chamber and 25 μL of cell suspension was added to the upper chamber of a 5-μm pore-sized chemoTX plate. After incubation at 37 °C under 5% CO2 atmosphere for 90 min, migrated cells were collected and cell populations were determined by surface expression of stained antibody in each population using a Gallios flow cytometer (Beckman coulter). Data are expressed as a migration index (number of migrated cells in the presence of chemoattractant divided by the cell number in the absence of chemokine).

### Flow cytometry

Cells for flow cytometric analysis were prepared as follows: bone marrow cells were flushed from femurs, followed by incubation with ACK buffer to remove red blood cells. Lung cells were prepared from the right inferior lobe by enzymatic digestion with 300 U mL^–1^ Type I collagenase (Sigma–Aldrich) and 2 U mL^−1^ DNase I (Merck Millipore). Tumor-associated cells were isolated by enzymatic digestion of tumor tissues as for lung cells, removal of debris using a Percoll density gradient, and lysis of red blood cells. Cells were washed and resuspended in PBS supplemented with 2% fetal calf serum and filtered through a 70-μm strainer. After blocking Fc receptors through incubation with an anti-mouse CD16/32 antibody (clone 2.4G2, dilution 1:100, Cat BE0307), cells were stained with fluorescence-labeled anti-mouse monoclonal antibodies. The antibodies used in these studies were CD45-FITC (clone 30-F11, dilution 1:400, Cat 103108), CD11b-Pacific Blue, CD11b-Brilliant Violet 605 or CD11b-FITC (clone M1/70, dilution 1:400, Cat 101224, 101257 and 101206), CD11c-APC-Cy7 (clone N418, dilution 1:400, Cat 117324), Ly-6C-APC-Cy7 (clone HK1.4, dilution 1:400, Cat 128026), Ly-6G-Alexa Fluor 700 (clone 1A8, dilution 1:200, Cat 127622), CD206-Alexa Fluor 647 (clone C068C2, dilution 1:200, Cat 141734), I-A/I-E- PerCP-Cy5.5 (clone M5/114.15.2, dilution 1:400, Cat 107626), CD24^−^FITC (clone M1/69, dilution 1:200, Cat 553261), F4/80-PE-Cy7 (clone BM8, dilution 1:400, Cat 123114), CD4-FITC (clone RM4-5, dilution 1:200, Cat 100510), B220-PE-Cy7 (clone RA3-6B2, dilution 1:200, Cat 103222), NK1.1-PerCP-Cy5.5 (clone PK136, dilution 1:200, Cat 108728), CD8-Pacific Blue or CD8-PerCP-Cy5.5 (clone 53-6.7, dilution 1:200 Cat 100725, 100734), CD80-APC (clone 16-10A1, dilution 1:200, Cat 104714), CD86-PE/Cy7 (clone GL-1, dilution 1:200, Cat 105014), CCR2-Alexa Fluor 647 (clone 475301, dilution 1:100, FAB5538R-025), CCR5-biotin (clone C34-3448, dilution 1:100, Cat 559922) and streptavidin-PE-Cy7 (1:200, Cat 405206). To stain Granzyme B, cells were incubated in cytofix/cytoperm fixation/permeabilization solution (BD biosciences), followed by staining with Granzyme B-Alexa Fluor 647 (clone GB11, dilution 1:100, Cat 515406). Dead cells were excluded through propidium iodide staining or Zombie Aqua staining (BioLegend). Flow cytometry data were obtained using a Gallios flow cytometer (Beckman Coulter) and analyzed with FlowJo software (version 10; FlowJo, LLC). Cell numbers in the lung are expressed per lung inferior lobe; cell numbers in the tumor are expressed per mg of tissue. For the detection of FROUNT promoter-driven GFP expressing (FROUNT-GFP) cells, B16 or LLC tumor cells were inoculated subcutaneously into FROUNT-GFP reporter mice. Tumor-infiltrating cells and bone marrow cells were measured for their expression of GFP along with the surface markers. Relative MFI of GFP channel was calculated by subtracting MFI in GFP channel of wild-type cells, which does not express GFP but were surface stained in an exactly the same way to FROUNT-GFP reporter mice. Gating strategies to identify the immune cell populations described in Figs. 3, 4, 7 and Supplementary Figs. 4, 5, 6 are shown in Supplementary Fig. 10^22,54^.

### Morphological analysis of macrophages

Peritoneal macrophages were isolated from mice, seeded in the 8-well glass-based dishes (Life Technologies) and incubated overnight at 37 °C to allow the cells to adhere. After serum starvation for 6 h, cells were stimulated with chemokine at a concentration of 100 ng mL^–1^, then fixed with 4% paraformaldehyde (Wako) and permeabilized with 0.1% Triton X-100 (Wako) in PBS, followed by staining with phalloidin conjugated with Alexa Fluor 594 (Life Technologies). For inhibitor analysis, peritoneal macrophages were pre-incubated with 25 μM DSF or wortmannin for 1 h prior to chemokine stimulation. Tumor macrophages were isolated from collagenase digestion of tumor tissue as attached cells by washing non-adherent cells away and further culturing them in DMEM containing 10 nM M-CSF (BD Biosciences) and 10% serum. After co-culture with LLC tumor cells, confocal images were obtained using an SP5 confocal microscope (Leica Microsystems). Cells were classified as lamellipodial/round-shaped or filopodial based on their morphology and the ratio of each type in the visual field determined.

### Tumor cell co-culture with monocytes/macrophages

Monocytes were enriched from bone marrow cells by MACS negative selection for CD3, B220, Ly-6G, NK1.1, Ter119, CD49b, and c-kit. The percentage of Ly-6C^hi^ Ly-6G^−^ monocytes were >80%. Monocytes (1 × 10^5^) and 100 DsRed^+^ LLC tumor cells were co-cultured for 24 h. Proliferating tumor cell clusters containing more than 10 cells were enumerated by fluorescent microscopy. For flow cytometry analysis, cultured cells were harvested with trypsin-EDTA after 3 days of culture. Cells were stained with CD45-FITC (clone 102, BioLegend), then stained with annexin V-APC in binding buffer (BD biosciences). For detection of DNA synthesis, 5-ethynyl-2´-deoxyuridine (EdU) was added to the culture in the final hour of incubation. Intracellular EdU was stained using Click-iT^®^ EdU Alexa Fluor™ 647 flow cytometry assay kits (Thermo Fisher Scientific) according to manufacturer’s instructions. Flow cytometry data were obtained by gating CD45(−) DsRed(+) cells and analyzing annexin V-positive cells or EdU-positive cells. Dead cells were excluded by Zombie Aqua staining (BioLegend). Stained cells were measured using Flow-Count™ Fluorospheres (Beckman Coulter) for the determination of absolute counts.

### High-throughput screening and validation

A small compound library of 131,200 compounds supplied as 10 or 2 mM solutions in DMSO was obtained from the University of Tokyo Drug Discovery Initiative (Tokyo, Japan) and screened using homogeneous time-resolved FRET (HTRF). For the HTRF assay in white 384-well low-volume microplates (Corning), 4 μL 20 nM GST-fused FROUNT (FNT-C)^25^, DMSO, or test compounds (2 μM or as indicated) were mixed in binding buffer [10 mM HEPES pH 7.4, 0.2 M potassium fluoride, 10 mM NaCl, 0.1% Tween 20, and 0.5% bovine serum albumin (BSA)] and incubated for 30 min. After incubation, 250 nM final concentration of biotinylated Pro-C peptide (corresponding to the FROUNT-binding region of CCR2 [310–325: EKFRRYLSVFFRKH] or CCR5 [302–317: EKFRNYLLVFFQKHIA])^25^, 2.6 ng anti-GST antibody labeled with Europium cryptate (Cisbio), and 12.5 ng high-grade XL665-conjugated streptavidin (Cisbio) were added to each well. After 20 h incubation at RT, HTRF signals were measured using an Envision reader (Perkin Elmer) at the emission wavelengths of 620 nm for the donor and 665 nm for the acceptor. The compounds with >30% inhibition for FROUNT-CCR2 interaction were tested for reproducible inhibition in the secondary assay, then tested for inhibition of an unrelated peptide-protein interaction control, a p53 peptide^55^ and GST-fused MDM2 (Abnova) protein. Nutlin^27^ was used as a selective inhibitor for this interaction. The interaction signal was calculated as follows: Ratio = Emission at 665 nm/Emission at 620 nm. Results are expressed as Delta F (%) value calculated as follows: Delta F (%) = 100 × (Sample Ratio – Ratio of negative control)/Ratio of negative control.

### Surface plasmon resonance

Interactions between FROUNT and library compounds were analyzed by surface plasmon resonance using a Biacore T100 (GE Healthcare). Full-length FROUNT protein or the FROUNT-binding region of CCR2 was immobilized on the CM5 sensor chip^56^. Library compounds (20 μM), DSF or DDC (concentrations as indicated) in HBS-EP buffer (GE Healthcare) were applied to the sensor chip with a flow rate of 30 μL per min in HBS-EP buffer containing 2% DMSO. The resonance unit (RU) was measured during the binding and washing periods, and the binding kinetics were analyzed. Solvent correction with DMSO was performed using Biacore T100 evaluation software.

### PH-Akt domain translocation assay

CCR2-expressing Chinese hamster ovary cells were transfected with PH-Akt-tHcRed. They were serum starved in the presence or absence of 25 μM DSF for 30 min, stimulated with CCL2 for 1 min, and then fixed with 4% PFA. Cells were visualized with confocal microscopy using SP5 (Leica).

### ALDH activity assay

THP-1 cells were incubated for 30 min with medium containing DMSO or inhibitors at the concentration of 33 μM then washed and incubated with ALDEFLUOR reagent (STEMCELL Technologies) in assay buffer according to the manufacturer’s instruction. Fluorescence derived from substrate metabolized by cellular ALDH were measured by Gallios cytometer.

### NMR spectroscopy

All ^1^H–^15^N heteronuclear single quantum correlation (HSQC) NMR spectra were measured at 25 °C on a Bruker 600-MHz AVANCE III spectrometer with a cryogenic probe. The titration was performed in 20 mM Tris–HCl buffer (pH 7.5, 50 mM NaCl, prepared in 95% H_2_O and 5% D_2_O at a final concentration of 12.5–50 µM DSF or DDC) and 50 µM CRBD of FROUNT (a.a. 532–656) bearing the L538E/P612S mutations or its mutant (C603S). The assignments of the backbone ^1^H^N^ and ^15^N NMR signals of CRBD were performed, based on the previous paper^30^. The NMR signal assignments of CRBD after the titration of DSF were separately performed (manuscript in preparation). Combined chemical shift differences Δδ was calculated by Δδ = [(Δδ_HN_)^2^ + (Δδ_N_/6.5)^2^]^1/2^, where Δδ_HN_ and Δδ_N_ are the chemical shift differences for ^1^H^N^ and ^15^N, respectively.

### Cytotoxicity and growth assays

Cytotoxicity was evaluated using an LDH cytotoxicity detection kit (TaKaRa) according to the manufacturer’s instructions. Briefly, culture supernatant from tumor cells was harvested after 48 h culture in the presence or absence of DSF, 5-FU (Kyowa Hakko Kirin), or IKK-IV (Merck Millipore) and assayed for the concentration of lactic acid released from damaged cells. To evaluate the effects of compounds on cell growth, cells were incubated with inhibitors for 48 h, then WST-1 (Dojindo) was added to the culture for the final 30 min. An Envision instrument (PerkinElmer) was used to measure the absorbance of each well at 450 nm versus a 650-nm reference to detect the amount of formazan produced from WST-1 by mitochondrial dehydrogenases in viable cells.

### Quantification of mRNA levels using real-time PCR

Total RNA was isolated using RNA-Bee regent (Tel-Test, Inc.) according to the manufacturer’s instructions. cDNA was generated from 1 μg total RNA using a SuperScript synthesis kit (Life Technologies). For real-time quantitative PCR, cDNAs were amplified using SYBR Green PCR Master Mix (Qiagen), with forward and reverse primers at a final concentration of 0.5 µM and in a sample volume of 20 µL. Assays were performed in duplicate using an ABI 7500 real-time PCR system or QuantStudio6 (Thermo Fisher Scientific) according to the manufacturer’s instructions. Results were normalized according to the expression levels of *Hprt* RNA. The primers used were as follows: *Hprt*, forward 5′-GGCAGTATAATCCAAAGATGGTCAA-3′ and reverse 5′-GTCTGGCTTATATCCAACACTTCGT-3′; *Frount*, forward 5′-TGATCGACTGACGTTTCTGG-3′ and reverse 5′-GAGAAGCAGCGTCACCAA A-3′; *Ccl2*, forward 5′-CATCCACGTGTTGGCTCA-3′ and reverse 5′-GATCATCTTGCTGGTGAATGAGT-3′; *Ccl7*, forward 5′-TTCTG TGCCTGCTGCTCATA-3′ and reverse 5′-GATCATCTTGCTGGTGAATGAGT-3′; *Ccl3*, forward 5′-GCTGTTCTTCTCTGTACCATGACAC-3′ and reverse 5′-TCAACG ATGAATTGGCGTG-3′; *Ccl4*, forward 5′-GATGGATTACTATGAGACCAGCAGTC-3′ and reverse 5′-CAACTCCAAGTCACTCATGTACTCAG-3′; and *Ccl5*, forward 5′-CATATGGCTCGGACACCACT-3′ and reverse 5′-ACACACTTGGCGGTTCCTTC-3′. Relative mRNA levels were determined by using the equation 2^−*ΔΔ*Ct^ relative to wild-type controls, after which data were transformed to log2 values.

### Immunohistochemical staining

Mice were anesthetized and perfused with PBS and the left lungs were isolated. The lungs were infused endotracheally with Optimal Cutting Temperature compound (OCT) (Sakura Finetechnical) and then embedded in OCT and frozen with liquid nitrogen. Fresh-frozen tissue sections were prepared (8-μm thickness) and fixed with 4% paraformaldehyde-PBS. After washing with 0.05% Tween 20 PBS, sections were blocked with Blocking One reagent (Nacalai Tesque) and stained with an anti-mouse F4/80 antibody (clone BM8, BioLegend) followed by staining with Alexa Fluor 488 anti-rat IgG (Life Technologies). Fluorescence images derived from B16-DsRed cells and stained antibodies were obtained using an SP5 confocal microscope (Leica Microsystems). The F4/80 + macrophage number accumulated in and around the tumor nodule visualized by fluorescent protein was counted per nodule of similar sizes among the control and FROUNT-cKO, vehicle, or DSF-treated groups.

### Chemotaxis assay using TAXIScan technology

The TAXIScan device (ECI, Tokyo, Japan) is an optically accessible horizontal chemotaxis apparatus consisting of an etched silicon substrate and a flat glass plate, which form two compartments separated by a 5-μm deep microchannel^57^. THP-1 cells were applied through a hole connected to one of the compartments and aligned along the start line on the edge of the channel. Human CCL2 (10 ng mL^–1^) was applied through a hole connected to the other compartment to form a concentration gradient in the channel. Time-lapse images were recorded every 30 s for 30 min and cell movement was analyzed using TAXIScan analyzer software (ECI, Tokyo, Japan).

### Statistical analyses

Each experiment was repeated at least three times except for clinical data. Statistical analyses were performed using GraphPad Prism software. Statistical significance was calculated using two-tailed unpaired *t*-tests or one-way ANOVA with post-hoc Tukey’s multiple comparison test. *P* < 0.05 was considered statistically significant. Results from all mice were included in the final analysis without exclusion. All graphs show mean values ± s.e.m.

### Reporting summary

Further information on research design is available in the Nature Research Reporting Summary linked to this article.

## Supplementary information

Supplementary Information

Reporting Summary

## Data Availability

The datasets for analysis of human transcriptome were downloaded from the following link: PrognoScan (http://dna00.bio.kyutech.ac.jp/PrognoScan/), Query: NUP85, downloaded on 2018/02/01; PRECOG, https://precog.stanford.edu/, downloaded on 2018/03/15, the tab-delimited (PCL) file, PRECOG_individZ.pcl; Human Protein Atlas, https://www.proteinatlas.org/, Query: NUP85, Kaplan-Meier-plot images obtained from “pathology” tab. RNA-seq reads in FASTQ format has been deposited in the NCBI Short Read Archive (SRA) under the BioProject accession number PRJNA588875. follows:[{https://www.ncbi.nlm.nih.gov/bioproject/PRJNA588875]. NMR data were deposited in the Protein Data Bank under the accession codes 6L5C. The remaining data in support of the findings of this study are available in the Article, Supplementary files or available from the corresponding author, Yuya Terashima, tera@rs.tus.ac.jp, upon reasonable request.
