# Supplementary material for: Targeting FROUNT with disulfiram suppresses macrophage accumulation and its tumor-promoting properties
Source: Nat Commun. 2020 Jan 30;11:609. doi: 10.1038/s41467-020-14338-5 (PMC6992764; doi:10.1038/s41467-020-14338-5)
Supplement: Supplementary file 1 — Supplementary Information [file 41467_2020_14338_MOESM1_ESM.pdf]

Supplementary information for

Targeting FROUNT with disulfiram suppresses macrophage accumulation and its tumor-promoting properties

Terashima Y. et al.

Supplementary Figure 1

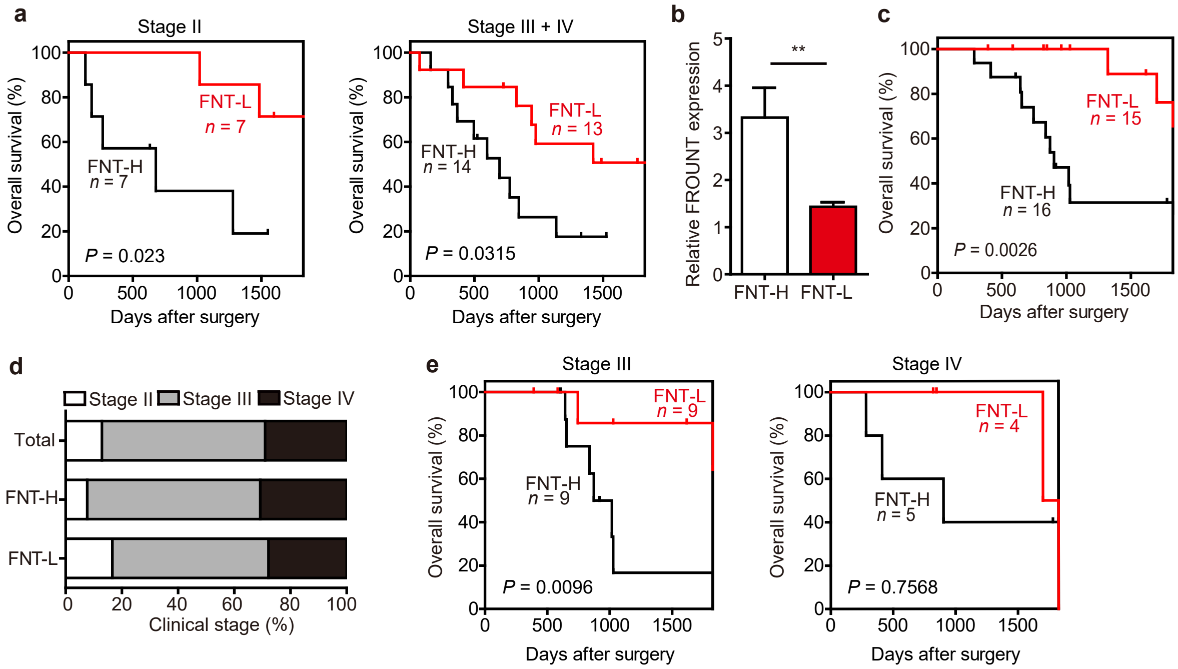

Supplementary Fig. 1 Retrospective analysis of resected lung cancer specimens. (**a**) Overall survival curves for the 40 patients separated by tumor stage described in **Figure 1**. (**b-e**) Thirty-one patients with lung cancer independent of the 40 patients analyzed in (**a**) and **Figure 1a-e** were divided into *FROUNT*-high (FNT-H) (n = 16) and *FROUNT*-low (FNT-L) (n = 15) groups (**b**), based on the relative expression of *FROUNT* mRNA in lung specimens (bars indicate median values). Overall survival curves estimated by Kaplan-Meier analysis and the log-rank test (**c**) and the proportion of patients in each stage (**d**). Overall survival curves of patients separated by tumor stage (**e**).**P < 0.01, by two-tailed, unpaired Student' s t-test. Error bars indicate s.e.m.

Supplementary Figure 2

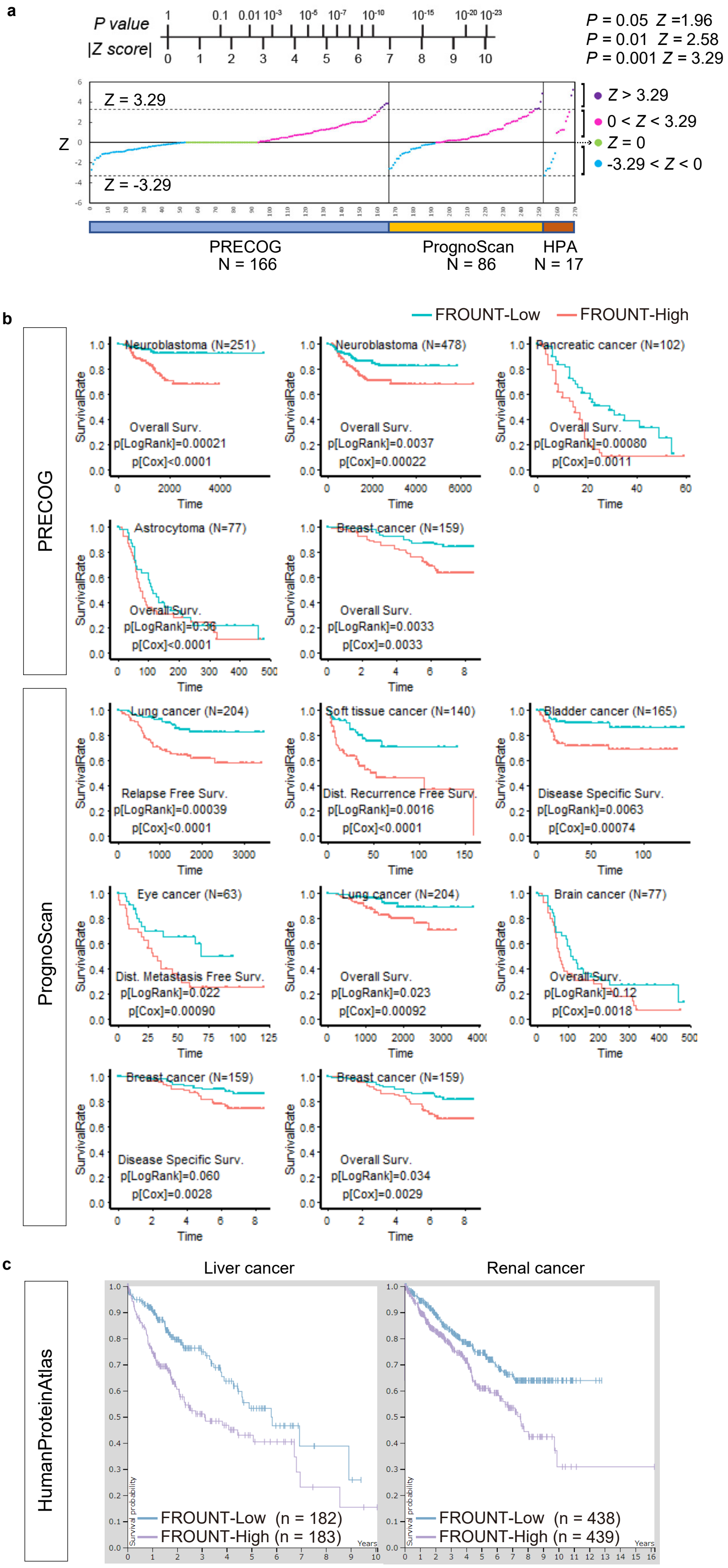

	Liver cancer	Renal cancer
Expression cutoff	5.6 FPKM	5.4 FPKM
5-year survival high	40%	61%
5-year survival low	53%	75%
Log-rank P value	4.98e-4	1.94e-4

Supplementary Fig. 2 Association of FROUNT with survival in various cancer. (**a**) Distribution of *Z* value for studies used for the survival analysis from human transcriptome databases: PRECOG (166 studies), PrognoScan (86 studies) and the Human Protein Atlas (17 studies). Studies with $Z \geq 3.29$ (corresponding to $P = 0.001$) for (**b**) were separately colored. (**b**) Survival analysis from human transcriptome databases: PRECOG (166 studies) and PrognoScan (86 studies). Datasets were selected using the FDR (Benjamini & Hochberg) method where $q < 0.05$ (the *q*-value is a measure of the strength of an observed statistic with respect to FDR20). For each dataset, Cox hazard model (*p*[Cox]) was used for evaluating the association of *FROUNT* expression on survival. Patients were divided into two groups based on median *FROUNT* expression, then log-rank tests (*p*[LogRank] was used to compare survival between the *FROUNT*-high (red line) and low groups (blue line). (**c**) Survival analysis from the Human Protein Atlas (17 studies) in which patients were divided based on level of *FROUNT* mRNA expression into *FROUNT*-high (red line) and -low groups (blue line) using median separation.

Supplementary Figure 3

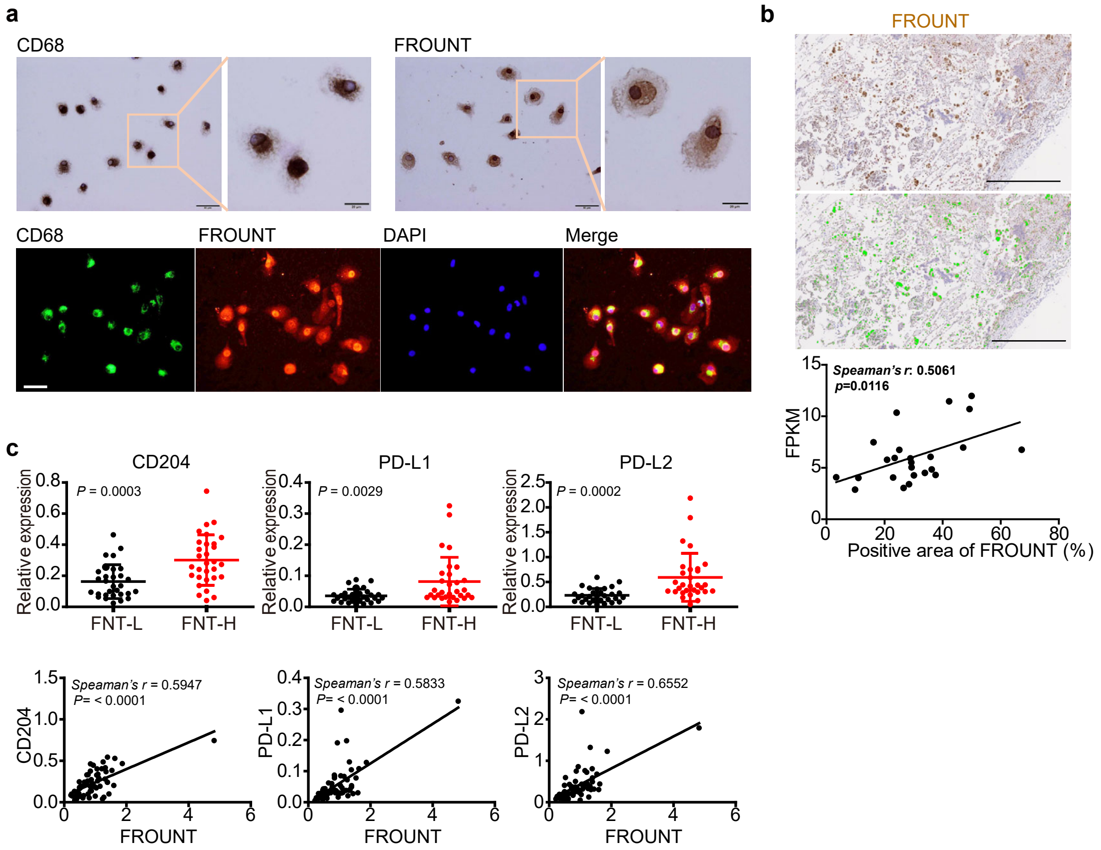

Supplementary Fig. 3 FROUNT expression in macrophages and association of FROUNT with myeloid cell-related immunosuppressive markers. (**a**) Immunostaining (upper panel) of FROUNT and CD68 (brown) and immunofluorescence staining (lower panel) of CD68 (green) and FROUNT (red) of human peripheral blood mononuclear cell-derived macrophages. Cells were counterstained with DAPI (blue). Scale bars: 50 μm. (**b**) FROUNT staining in a representative case with non-small-cell lung cancer (NSCLC) (upper panel) and a WinRoof digital image, with green corresponding to the area of FROUNT-positive staining (lower panel). Statistical dot plots showing correlation between positive area of FROUNT and FPKM value for FROUNT in RNAseq data. Scale bars, 500 μm. (**c**) Comparison of mRNA expression of myeloid cell-related immunosuppressive markers between *FROUNT*-high (FNT-H) and -low (FNT-L) group in lung adenocarcinoma patients based on qPCR data of lung cancer specimen. Pooled data from two cohorts in **Figure 1** and **Supplementary Fig. 1** were shown.

Supplementary Figure 4

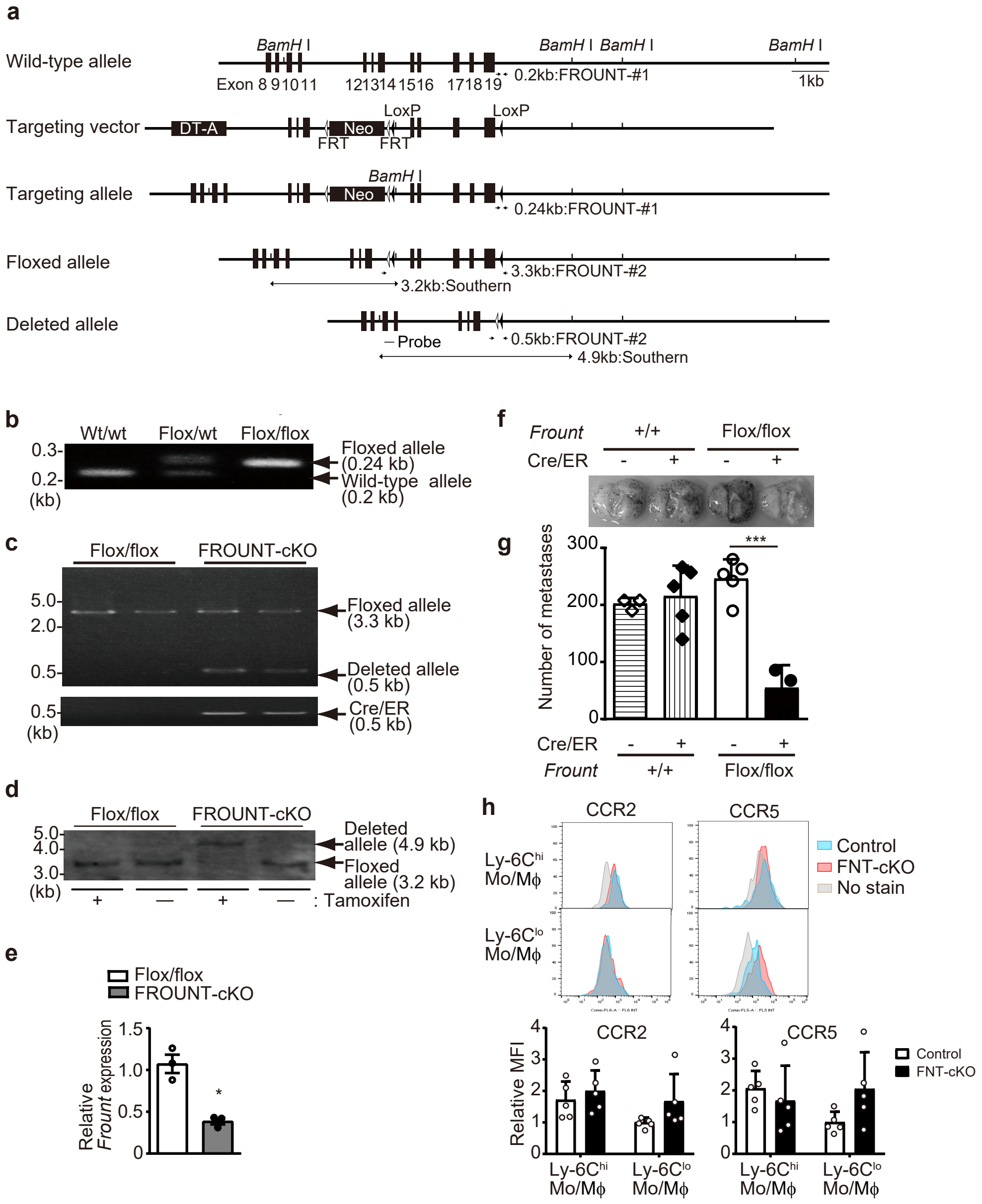

Supplementary Fig. 4 Generation of *Frount*-conditional knockout (FROUNT-cKO) mice. (**a**) Gene-targeting strategy to generate a floxed allele for murine *Frount*. DT-A, diphtheria toxin A selection cassette; Neo, neomycin-resistance cassette. (**b-d**) Homozygous *Frount*-floxed mice were generated and crossed with CAG-Cre/ER transgenic mice. (**c,d**) Tamoxifen treatment successfully induced gene recombination and deletion of the floxed allele. (**b,c,** Genomic PCR; **d**, Southern blot). (**e**) The expression of *Frount* mRNA in FROUNT-cKO mice was reduced to less than 30% of that in the control *Frount*-floxed mice. (**f,g**) The effect of Cre expression on tumor progression, wild-type mice and CAG-Cre/ER transgenic mice without any floxed allele, and homozygous *Frount*-floxed mice with or without Cre/ER were administrated with tamoxifen and injected intravenously with B16 melanoma cells. Mice were sacrificed on day 11 after tumor cell injection for enumeration of the lung metastatic nodules. Representative images of (**f**) tumor-bearing lungs, and (**g**) number of lung metastatic nodules (+/+_Cre-: $n = 3$, +/+_Cre+: $n = 5$, Flox/flox_Cre-: $n = 5$, Flox/flox_Cre+: $n = 3$, means ± s.e.m.). Reduced metastasis was only observed in the presence of the *Frount*-floxed allele and Cre/ER expression. (**h**) Expression of CCR2 and CCR5 on tumor-associated Ly-6C^{hi} or Ly-6C^{lo} monocytes/macrophages (Mo/Mϕ) accumulated in subcutaneous B16 tumors grown in control (LysMCre) or FROUNT-cKO mice ($n = 5$). *$P < 0.05$, by two-tailed, unpaired Student' s t-test. ***$P < 0.001$, by one-way ANOVA with Tukey' s multiple comparison test. Error bars indicate s.e.m.

Supplementary Figure 5

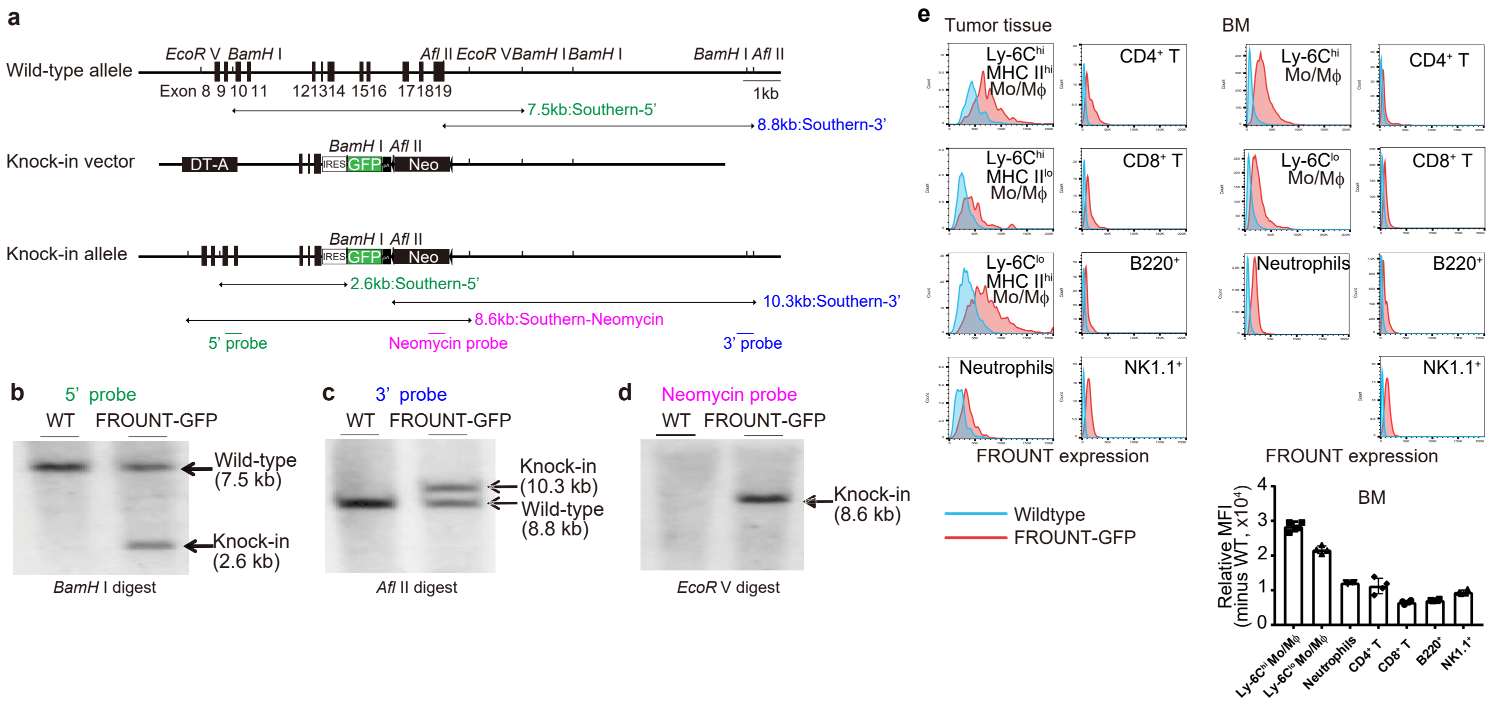

Supplementary Fig. 5 Generation of FROUNT reporter (FROUNT-GFP) mice. (**a**) Gene-targeting strategy to generate FROUNT reporter mice expressing GFP under the control of a *Frount* promoter. Heterozygous *Frount-gfp*-knock-in (FROUNT-GFP) mice were generated. DT-A, diphtheria toxin A selection cassette; IRES, internal ribosome entry site; Neo, neomycin-resistance cassette. (**b-d**) Detection of the knock-in allele in *Frount-gfp-knock*-in mice by Southern blot analysis with specific probes (**b**, 5'-probe; **c**, 3'-probe; and **d**, neomycin probe). (**e**) Representative histogram plots of **Fig. 3a** showing FROUNT (GFP) protein expression in tumor-infiltrating cells, and bone marrow cells (monocytes/macrophages subsets; Ly-6C^{hi} and Ly-6C^{lo}, Neutrophils, CD4/8 T cells, B cells and NK cells) of FROUNT-GFP reporter mice as measured by flow cytometry (red line). Background signal of cells from wild-type mice was overlaid (blue line). Mean fluorescence intensity data of bone marrow cells subtracting those of wild-type cells were plotted (n = 5).

Supplementary Figure 6

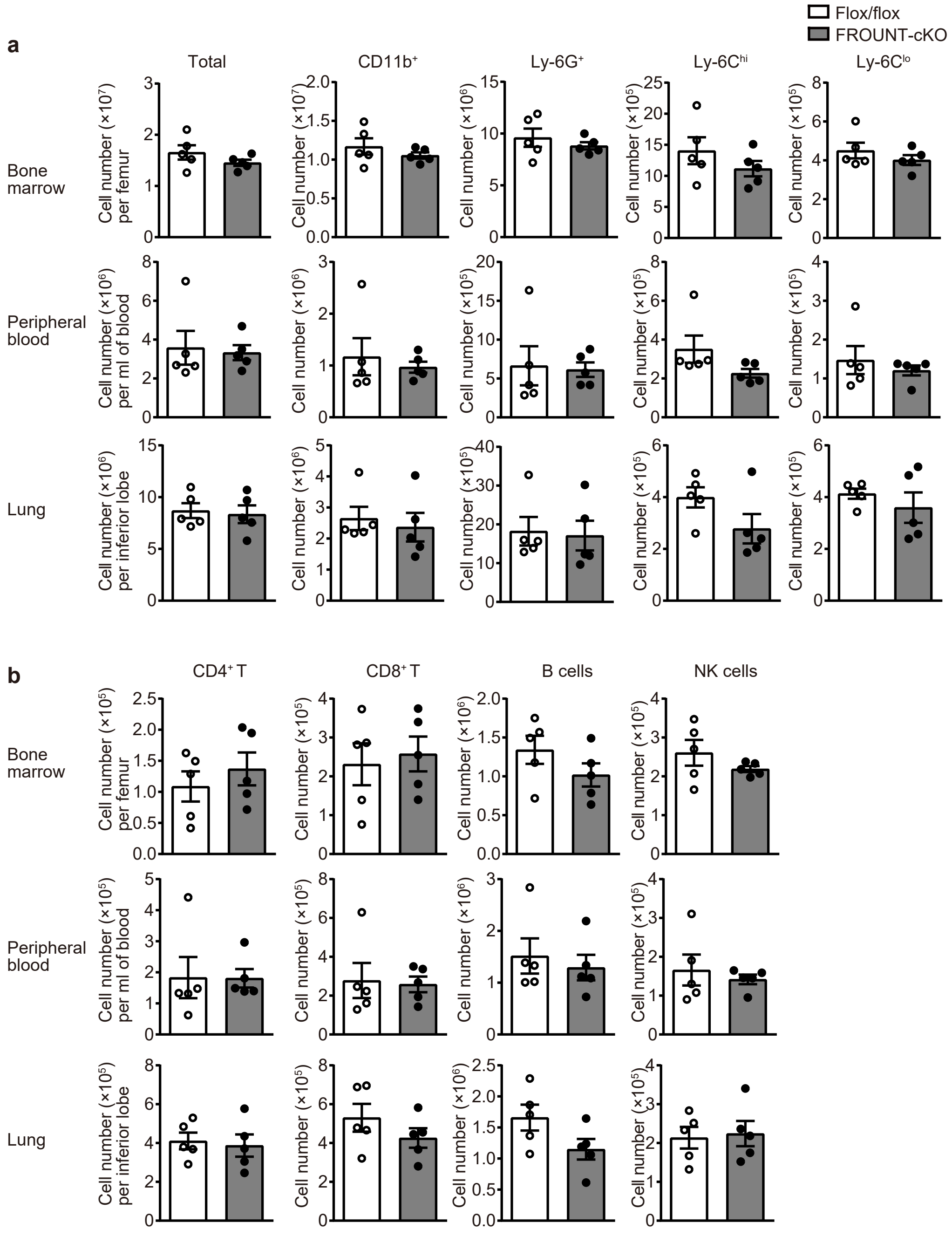

Supplementary Fig. 6 *Frount*-deficiency does not alter leukocyte subsets in the steady state. Myeloid subsets (**a**) and lymphocyte subsets (**b**) in *Frount*-floxed (white bars) or FROUNT-cKO (gray bars) mice (n = 5). Cells were isolated from the bone marrow, peripheral blood, and lung, and stained with surface markers of myeloid (Ly-6G⁺ neutrophils and Ly-6C$^{hi/lo}$ macrophages in CD11b⁺ cells) and lymphocyte (CD4⁺ and CD8⁺ T cells, B220⁺ B cells, and NK1.1⁺ natural killer (NK) cells) subsets; the numbers of each subset were analyzed by flow cytometry (means ± s.e.m.).

Supplementary Figure 7

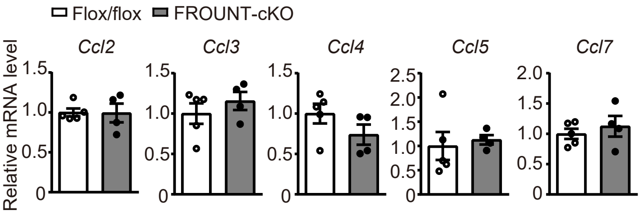

Supplementary Fig. 7 *Frount*-deficiency does not alter expression of chemokines in tumor-bearing lungs. mRNA expression of macrophage-recruiting chemokines in tumor-bearing lungs from *Frount*-floxed (white bars) or FROUNT-cKO (gray bars) (Flox/flox: n = 5, cKO: n = 4 per group; representative of 3 independent experiments).

Supplementary Figure 8

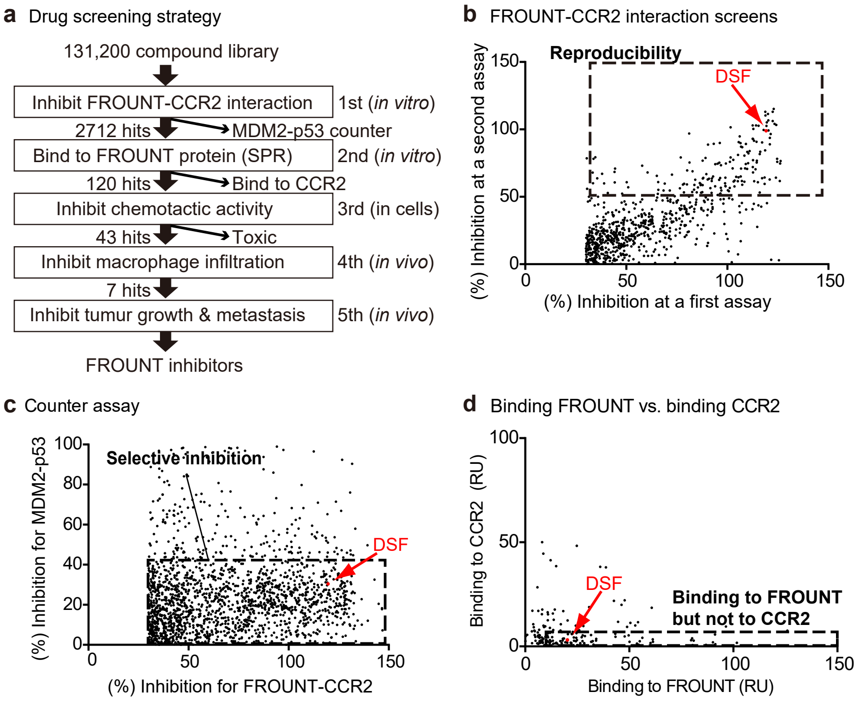

Supplementary Fig. 8 Multi-step screening strategy used to identify FROUNT inhibitors. (**a**) Library compounds (131,200 in total) were tested for inhibition of the FROUNT-CCR2 interaction, using a homogenous time-resolved FRET (HTRF) screening assay and then 2,712 selected compounds were tested for binding to the FROUNT protein by surface plasmon resonance (SPR) *in vitro*. Selected 120 compounds were tested in cell-based assays for chemotaxis inhibition without cytotoxicity, assessed for the ability to inhibit macrophage infiltration *in vivo* (43 compounds), and finally 7 hits were tested for anti-tumor activity in mouse tumor growth and metastasis models. (**b,c**) The results of primary screening showing the percent inhibition values (FROUNT-CCR2 interaction) for each compound in the first assay versus the second assay (**b**), and versus the counter assay (MDM2-p53 interaction) (**c**). (**d**) Screening of primary hit compounds for binding to FROUNT in SPR. Plots of DSF are shown in red.

Supplementary Figure 9

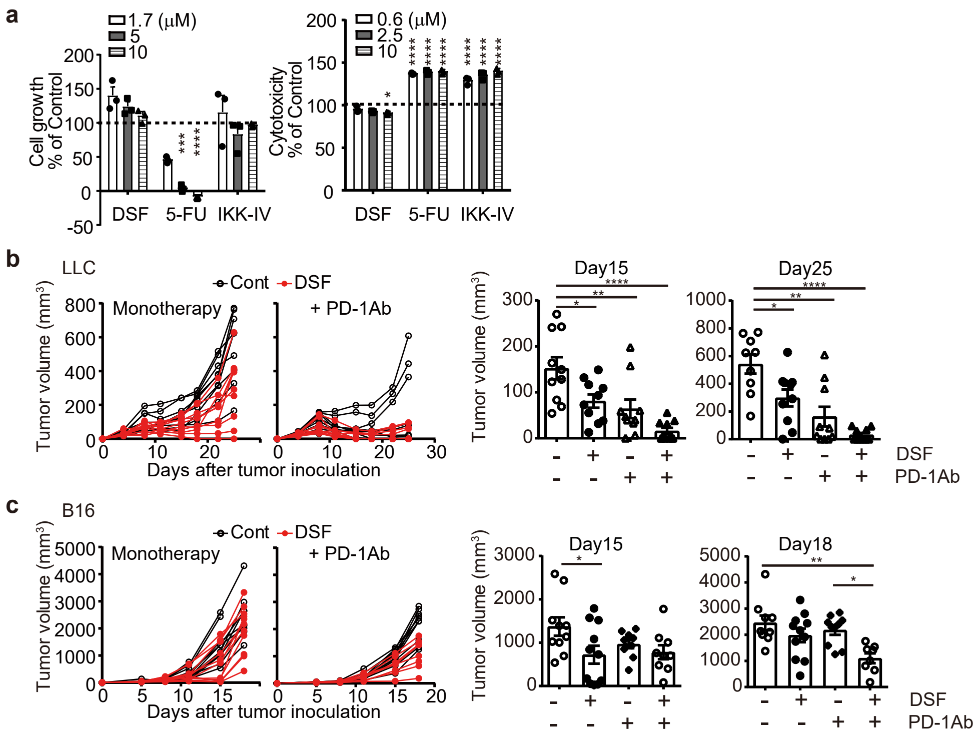

Supplementary Fig. 9 DSF has negligible direct effects on tumor cell growth and viability, but exerts synergistic effect with the immune checkpoint inhibitor, anti-PD-1 antibody, to inhibit *in vivo* subcutaneous tumor growth. (**a**) Effects on B16 tumor cell growth (left) and cytotoxicity (right) in 48-h culture in the presence of DSF, the cytotoxic anti-cancer drug 5FU, and the NF-κB inhibitor IKK-IV, as measured by the WST-1 assay and the LDH cytotoxicity assay, respectively (means ± s.e.m., n = 3, s.e.m for the control in WST-1 assay = 1.156 and for the control in LDH assay = 0.7489, data representative of two independent experiments). (**b,c**) Effects of combination treatment with DSF and an anti-PD-1 antibody in LLC and B16 subcutaneous tumor models, as described in **Figure 8a-d**. Tumor growth curves (left) for individual mice of (**b**) LLC and (**c**) B16 and tumor volume (right) on day 15 for LLC and B16, on day 25 for LLC and on day 18 for B16 (n = 9). *P < 0.05, **P < 0.01, ***P < 0.001, ****P < 0.0001 by one-way ANOVA with Tukey’ s multiple comparison test.

Supplementary Figure 10

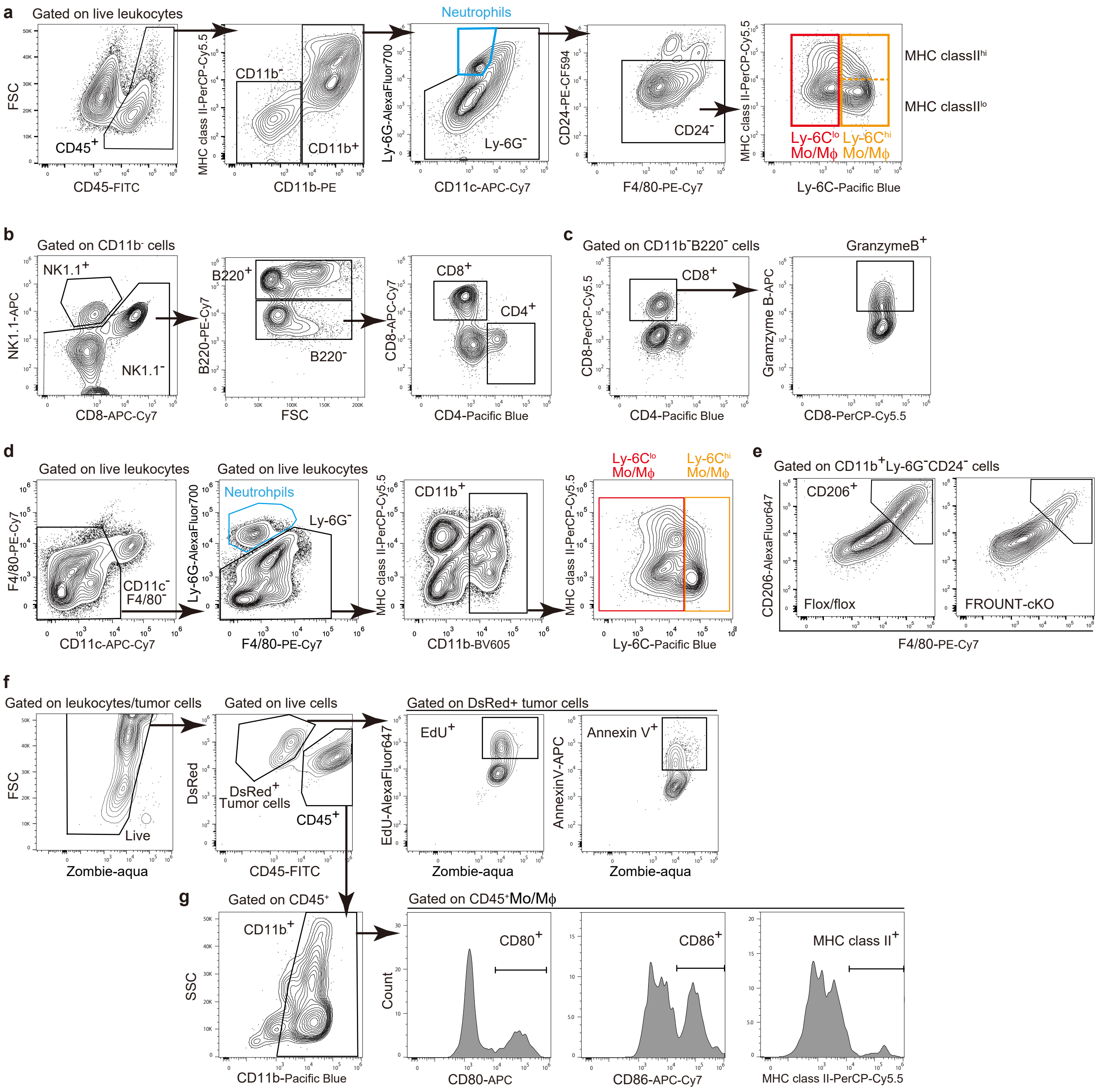

Supplementary Fig. 10 Flow cytometry gating strategies used to identify immune cell populations. (**a–e**) Representative plots for neutrophils and Ly-6C^{hi} and Ly-6C^{lo} monocytes/macrophages (Mo/Mϕ) (**a**), NK1.1^{+} cells, B cells, and CD4^{+} and CD8^{+} T cells (**b**) for Fig. 3a, b, Fig. 7c, and Supplementary Fig. 4h, 5e and Granzyme B^{+} CD8 T cells for Fig. 8d (**c**) in tumor tissue and lung neutrophils and Ly-6C^{hi} and Ly-6C^{lo} monocytes/macrophages for Fig. 3d (**d**), as well as CD206^{+} macrophages in tumor tissue for Fig. 3b and Fig. 7c, with representative plots from Frount-flox/flox and FROUNT-cKO mice (**e**). (**f, g**) Representative plots of EdU^{+} and Annexin V^{+} tumor cells in monocyte–tumor cell co-culture experiments for Fig. 4d (**f**) and expression of activation markers on monocytes/macrophages (**g**) for Fig. 4b, c, e.
