# Supplementary material for: Targeting FROUNT with disulfiram suppresses macrophage accumulation and its tumor-promoting properties
Source: Nat Commun. 2020 Jan 30;11:609. doi: 10.1038/s41467-020-14338-5 (PMC6992764; doi:10.1038/s41467-020-14338-5)
Supplement: Supplementary file 2 — Reporting Summary [file 41467_2020_14338_MOESM2_ESM.pdf]

nature research

Corresponding author(s): Dr Yuya Terashima

Last updated by author(s): Nov 26, 2019

Reporting Summary

Nature Research wishes to improve the reproducibility of the work that we publish. This form provides structure for consistency and transparency in reporting. For further information on Nature Research policies, see Authors & Referees and the Editorial Policy Checklist .

Statistics

For all statistical analyses, confirm that the following items are present in the figure legend, table legend, main text, or Methods section.

n/a	Confirmed	
☐	☒	The exact sample size (*n*) for each experimental group/condition, given as a discrete number and unit of measurement
☐	☒	A statement on whether measurements were taken from distinct samples or whether the same sample was measured repeatedly
☐	☒	The statistical test(s) used AND whether they are one- or two-sided<br>*Only common tests should be described solely by name; describe more complex techniques in the Methods section.*
☒	☐	A description of all covariates tested
☒	☐	A description of any assumptions or corrections, such as tests of normality and adjustment for multiple comparisons
☐	☒	A full description of the statistical parameters including central tendency (e.g. means) or other basic estimates (e.g. regression coefficient) AND variation (e.g. standard deviation) or associated estimates of uncertainty (e.g. confidence intervals)
☐	☒	For null hypothesis testing, the test statistic (e.g. *F*, *t*, *r*) with confidence intervals, effect sizes, degrees of freedom and *P* value noted<br>*Give P values as exact values whenever suitable.*
☒	☐	For Bayesian analysis, information on the choice of priors and Markov chain Monte Carlo settings
☒	☐	For hierarchical and complex designs, identification of the appropriate level for tests and full reporting of outcomes
☒	☐	Estimates of effect sizes (e.g. Cohen's *d*, Pearson's *r*), indicating how they were calculated

Our web collection on statistics for biologists contains articles on many of the points above.

Software and code

Policy information about availability of computer code

Data collection	Envision software (PerkinElmer), Kaluza Software (Beckmann Coulter), SP5 (Leica Microsystems), TAXIScan analyzer (ECI, Inc.), 7500 Software (Applied Biosystems)
Data analysis	Flow Jo software (BD Biosciences), Microsoft Excel (Office 2013), GraphPad Prism version 7 (GraphPad Software Inc.)

For manuscripts utilizing custom algorithms or software that are central to the research but not yet described in published literature, software must be made available to editors/reviewers. We strongly encourage code deposition in a community repository (e.g. GitHub). See the Nature Research guidelines for submitting code & software for further information.

Data

Policy information about availability of data

All manuscripts must include a data availability statement. This statement should provide the following information, where applicable:

- Accession codes, unique identifiers, or web links for publicly available datasets
- A list of figures that have associated raw data
- A description of any restrictions on data availability

All relevant data are available in the article and in the Supplementary data file. Any further detail is available from the corresponding author on reasonable request.

Field-specific reporting

Please select the one below that is the best fit for your research. If you are not sure, read the appropriate sections before making your selection.

☒ Life sciences ☐ Behavioural & social sciences ☐ Ecological, evolutionary & environmental sciences

Life sciences study design

All studies must disclose on these points even when the disclosure is negative.

Sample size	Sample size was determined based on the numbers reported in the field.
Data exclusions	No data were excluded.
Replication	All experiments were repeated multiple times as indicated in each figure legend
Randomization	To test the anti-tumor effects in a subcutaneous tumor growth model, mice were grouped into control and treatment groups based on their tumor size. For evaluation of anti-tumor effect of the inhibitor in spontaneous mammary tumor model, female MMTV-PyVT transgenic mice were randomly divided into two groups. Mice analyzed were litter mates and sex-matched whenever possible.
Blinding	Investigators were not blinded to mouse genotypes or treatments during experiments.

Reporting for specific materials, systems and methods

We require information from authors about some types of materials, experimental systems and methods used in many studies. Here, indicate whether each material, system or method listed is relevant to your study. If you are not sure if a list item applies to your research, read the appropriate section before selecting a response.

Materials & experimental systems

n/a	Involved in the study	
☐	☒	Antibodies
☐	☒	Eukaryotic cell lines
☒	☐	Palaeontology
☐	☒	Animals and other organisms
☐	☒	Human research participants
☒	☐	Clinical data

Methods

n/a	Involved in the study	
☒	☐	ChIP-seq
☐	☒	Flow cytometry
☒	☐	MRI-based neuroimaging

Antibodies

Antibodies used	The antibodies used in these studies were anti-PD-1 antibody (clone J43, BioXcell), CD16/32 antibody (BioXcell), CD45-FITC (clone 30-F11, BioLegend), CD11b-Pacific Blue or Brilliant Violet 605 (clone M1/70, BioLegend), CD11c-APC-Cy7 (clone N418, BioLegend), Ly-6C-APC-Cy7 (clone HK1.4, BioLegend), Ly-6G-Alexa Fluor 700 (clone 1A8, BioLegend), CD206-Alexa Fluor 647 (clone C068C2, BioLegend), I-A/I-E- PerCP-Cy5.5 (clone M5/114.15.2, BioLegend), CD24-FITC (clone M1/69, BD Biosciences), F4/80-PE-Cy7 (clone BM8, BioLegend), CD4-FITC (clone RM4-5, BioLegend), B220-PE-Cy7 (clone RA3-6B2, BioLegend), NK1.1-PerCP-Cy5.5 (clone PK136, BioLegend), CD8-Pacific Blue (clone 53-6.7, BioLegend), CD80-APC (clone 16-10A1, BioLegend) , CD86-APC/Cy7 (clone GL-1, BioLegend), Granzyme B-Alexa Fluor® 647 (clone GB11, BioLegend), CCR2-Alexa Fluor 647 (clone 475301, R&D systems) and CCR5-biotin (clone C34-3448, BD biosciences).
Validation	The antibodies have been validated by the vendors or by our previous published work.

Eukaryotic cell lines

Policy information about cell lines

Cell line source(s)	Lewis lung carcinoma (LLC), B16F10 (B16), THP-1 cells and CHO cells were obtained from the American Type Culture Collection.
Authentication	None of the cell lines used were authenticated.
Mycoplasma contamination	Cell lines were tested negative for mycoplasma.
Commonly misidentified lines (See ICLAC register)	No cell lines used are listed in the database of commonly misidentified cell lines.

Animals and other organisms

Policy information about studies involving animals; ARRIVE guidelines recommended for reporting animal research

Laboratory animals	Description of research mice used for experiments can be found in the relevant figure legends and Methods.

Wild animals	Not used.
Field-collected samples	Not used.
Ethics oversight	All animal studies were performed in accordance with the guidelines of the Animal Care and Use Committee of the University of Tokyo and Animal Care and Use Committee of Tokyo University of Science.

Note that full information on the approval of the study protocol must also be provided in the manuscript.

Human research participants

Policy information about studies involving human research participants

Population characteristics	This study consisted of 40 female patients with lung adenocarcinoma greater than stage II treated in the Department of Thoracic Disease, Chiba Cancer Center from 1997 to 2004.
Recruitment	Patients provided written informed consent to participate, then underwent complete resection of the lobe or segment in which the tumor resided.
Ethics oversight	The study was approved by the institutional review boards/ethics committees of the Chiba Cancer Center and the Research, Kumamoto University Hospital Review Board, Ethics Committee of the Faculty of Medicine, University of Tokyo, and Ethics Committee of Tokyo University of Science

Note that full information on the approval of the study protocol must also be provided in the manuscript.

Flow Cytometry

Plots

Confirm that:

- [x] The axis labels state the marker and fluorochrome used (e.g. CD4-FITC).
- [x] The axis scales are clearly visible. Include numbers along axes only for bottom left plot of group (a 'group' is an analysis of identical markers).
- [x] All plots are contour plots with outliers or pseudocolor plots.
- [x] A numerical value for number of cells or percentage (with statistics) is provided.

Methodology

Sample preparation	See methods
Instrument	Gallios
Software	FlowJo_VX
Cell population abundance	See methods
Gating strategy	See methods and supplementary figure

- [x] Tick this box to confirm that a figure exemplifying the gating strategy is provided in the Supplementary Information.
